# Host Transcriptional Regulatory Genes and Microbiome Networks Crosstalk through Immune Receptors Establishing Normal and Tumor Multiomics Metafirm of the Oral-Gut-Lung Axis

**DOI:** 10.3390/ijms242316638

**Published:** 2023-11-23

**Authors:** Beatriz Andrea Otálora-Otálora, Juan Javier López-Rivera, Claudia Aristizábal-Guzmán, Mario Arturo Isaza-Ruget, Carlos Arturo Álvarez-Moreno

**Affiliations:** 1 Grupo de Investigación INPAC, Unidad de Investigación, Fundación Universitaria Sanitas, Bogotá 110131, Colombia; claristizabal@unisanitas.edu.co; 2 Grupo de Investigación INPAC, Specialized Laboratory, Clinica Universitaria Colombia, Clínica Colsanitas S.A., Bogotá 111321, Colombia; jjlopez@colsanitas.com; 3 Keralty, Sanitas International Organization, Grupo de Investigación INPAC, Fundación Universitaria Sanitas, Bogotá 110131, Colombia; misaza@keralty.com; 4 Infectious Diseases Department, Clinica Universitaria Colombia, Clínica Colsanitas S.A., Bogotá 111321, Colombia; calvarez@colsanitas.com

**Keywords:** cancer, genomics, epigenomics, transcriptomics, transcriptional regulatory network (TRN), oral-gut-lung axis, host–microbiome crosstalk, adaptive and innate immune receptors

## Abstract

The microbiome has shown a correlation with the diet and lifestyle of each population in health and disease, the ability to communicate at the cellular level with the host through innate and adaptative immune receptors, and therefore an important role in modulating inflammatory process related to the establishment and progression of cancer. The oral cavity is one of the most important interaction windows between the human body and the environment, allowing the entry of an important number of microorganisms and their passage across the gastrointestinal tract and lungs. In this review, the contribution of the microbiome network to the establishment of systemic diseases like cancer is analyzed through their synergistic interactions and bidirectional crosstalk in the oral-gut-lung axis as well as its communication with the host cells. Moreover, the impact of the characteristic microbiota of each population in the formation of the multiomics molecular metafirm of the oral-gut-lung axis is also analyzed through state-of-the-art sequencing techniques, which allow a global study of the molecular processes involved of the flow of the microbiota environmental signals through cancer-related cells and its relationship with the establishment of the transcription factor network responsible for the control of regulatory processes involved with tumorigenesis.

## 1. Introduction

The human microbiome has been currently related to the early stages of inflammatory and oncogenic processes by inducing oxidative stress, genotoxicity, and host immune response disturbance [1]. About 2.2 million cancer cases have been attributed to microbial infection worldwide. The primary cancer causes were found to be related to infection with *Helicobacter pylori* (810,000 cases), human papillomavirus (690,000 cases), hepatitis B virus (360,000 cases), hepatitis C virus (160,000 cases), human herpesvirus-8 (KSHV/HHV8) (44,000 cases) [2], and Epstein–Barr virus (EBV) (357,900 cases) [3]. However, the human microbiome is composed of 500–1000 species of bacteria, viruses, and fungi that colonize the human body, including the mouth, lung, and gut, which can be more abundant than somatic cells, have more genes than our human genome, and interact synergistically to influence directly or indirectly important human physiological functions like nutrition, metabolism, immunity, and defense against pathogens [4]. The human bacterial microbiome, mycobiome, and virome are acquired during birth and constantly modeled throughout life by environmental factors. The oral cavity is the main entry of the microbiota and the second largest microbial habitat preceded by the gut; it is directly connected to the respiratory and the gastrointestinal tracts, allowing the translocation of disease-related microbiome profiles to the lung and gut due to oral–gut barrier dysfunction, altering its communication and the inter-connections with pulmonary and mucosal microbiomes [5]. The deviation of the balance between human microbiome synergistic interactions leads to “dysbiosis”, which enhances colonization, persistence, and pathogenicity [6]. As the bacteria increase in numbers geometrically as a result of binary fission and reach high density, large quantities of autoinducers are produced and are able to bind to the signaling receptors on the bacterial surface in sufficient quantity so as to activate the quorum-sensing genes that enable the bacteria to now behave as a multicellular population [7]. Therefore, polymicrobial synergy arises from physical interactions between microorganisms and host cells as well as the diffusion of soluble factors, which initiate cell-extrinsic and cell-intrinsic signals that are both able to modulate the gene expression of cancer-related cells, specifically triggering the activation and repression of the transcription factor network (TRN) or immediate-early genes (IEGs) network within minutes after stimulation without the need for de novo protein synthesis. Extracellular and intracellular signals are transduced from the cell membrane and endospores membrane through innate and adaptive immune receptors activating a chain of proteins in the cell within signaling pathways that will eventually activate and translocate transcription factors (TFs) to the nucleus and thereby induce or inhibit gene expression. The IEGs have essential biological roles in environmental signals and stress response; therefore, they might also have important roles in various diseases, including cancer development [8]. The crosstalk between host microbiome and transcriptional regulatory networks through immune receptors could be the essential channel of communication between genes and the environment to promote important changes in the growth, proliferation, differentiation, survival, and apoptosis mechanisms of cancer-related cells. In this review, we offer insights about the crosstalk between the microbiome and the host cells transcriptional networks through the host immune cells receptors by studying the results generated by high-throughput sequencing techniques in the global analysis of the multiomics regulatory processes (genomics, transcriptomics, and epigenomics) associated with the imbalance of the microbiota (metagenomics and metatranscriptomics), which may be involved in the acquisition of the hallmarks of cancer, in order to establish a baseline for future studies about the impact of the microbiota and host immune cells receptors related to an specific population in the tumorigenic processes connecting the oral-gut-lung axis.

## 2. The Molecular Metafirm of the Gut-Lung Axis during Tumorigenesis

In the postgenomic era, three important elements can be considered simultaneously for the elucidation of the etiology of complex diseases such as colorectum, gastric, and lung cancer, which cannot be contemplated in a traditional genetic approach. The first element is the large number of changes in the DNA sequence of genes (genomics) such as mutations, insertions, and deletions like ethnicity-related polymorphisms and haplotypes that have been associated with specific types of cancer involved in the ancestry of certain populations, which can now be studied all together to evaluate their added impact on the development of complex diseases such as cancer. The second element is the multiple regulatory processes involved in the establishment and progression tumorigenesis, which are related to epigenetic mechanisms like the formation of TFs complexes (transcriptomics), and the levels of methylation (epigenomics) that affect gene expression without changing the DNA sequence. Third is the great complexity, variability, and crosstalk between immune receptors of host and tumor cells and the environment through the microbiome network (metagenomics and metatranscriptomics) at the molecular level in each individual and every population, which leads to the application of multiomics studies, bioinformatics analysis, and artificial intelligence models able to predict specific interactions between the human transcriptome and microbiome networks for the developing of cancer and to characterize the intricate nature of host–microbiota interactions that might be related to the settlement of several immune, inflammatory, and oncogenic processes. 

### 2.1. Genomics Studies 

The evolution of the cancer genomics field is transforming the molecular characterization of a wide variety of different cancers. The application of next-generation sequencing technology for lung, colorectum, and gastric cancer has helped to better define the complex genomic landscape. Genomic studies have developed novel genomics-based molecular classification systems for cancers’ subtypes, supported by the classic driver mutations in cancer pathogenesis and leading to the discovery of new ones that previously were not known to be associated with the tumorigenic process. 

#### 2.1.1. Colorectum Cancer

Colorectum cancer is the third type of cancer with the highest incidence after breast and lung, and it is the second leading cause of cancer-associated deaths after lung worldwide [9]. Around 70% of colorectum cases are sporadic and are associated with environmental and dietary factors (cigarette smoking, excessive alcohol consumption, sedentary lifestyle, obesity, and diets high in fat and low in fiber intake). Around 25% of colorectum cases are familial and affect individuals with family history. Between 5 and 10% of colorectum cases are genetic or inherited cases and are categorized based on the presence or absence of colonic polyps [10]. During the initial stage from normal tissue to polyps to cancer (polyp size ≤ 2 cm), matrix metalloproteinases MMP1, MMP3, and MMP7 increase steadily, playing a role in cancer proliferation, invasion, and metastasis through the remodeling of extracellular matrix, which is strongly associated with poor prognosis [11,12]. *IL-8* or *CXCL8* also increases during the period of transition from normal tissue to polyp size ≤ 2 cm, while *CXCL7* increases at polyp size > 2 cm, suggesting that these could become potential cancer biomarkers of every tumor stage [13,14]. Meanwhile, the chemokines *CCL19* and *CXCL13* exhibit a rapid decline in cancer tissues, probably related to *CCL19* anti-angiogenic function in colorectum cancer [15,16]. The dynamic pattern of molecular interaction networks of the matrix metalloprotein family and chemokines during colon cancer progression provides relevant markers for more accurate screening of cancer in polyps [17]. 

The Cancer Genome Atlas (TCGA) project classified colorectum cancer in two molecular pathological subtypes according to array-based and sequencing technologies [18]. The first group has hypermutated tumors (~16%) with microsatellite instability (MSI) due to defective mismatch repair (~13%) or ultra-mutated tumors with DNA polymerase epsilon or delta 1 exonuclease domain (proofreading) mutations (~3%). The second group has non-hypermutated tumors (~84%), microsatellite stability (MSS) with a high frequency of DNA somatic copy number alterations, and dysregulated Wnt pathways with frequent mutations in genes including *APC*, *KRAS*, *PIK3CA*, *SMAD4*, and *TP53* [18,19,20]. The consensus is that molecular subtypes of colorectal cancer capture tumor heterogeneity at the gene-expression level, holding clear potential for clinical prediction, prognosis, and response to systemic therapy, which seems to be independent of the classifier used [21]. The classic model of progression from adenoma to colon carcinoma involves the inactivation of tumor-suppressor genes (*APC*, *TP53*, and *DCC*) and mutations in oncogenes (*KRAS* (40%), *SMAD*, and *BRAF* (10%)) [22] that lead to genomic instability [23]. *APC* (18.1%), *KRAS* (25%), and *TP53* (4.5%) have a lower frequency as compared to reports in studies from the United States and Europe (70%, 66%, and 60%) but a frequency similar to those reported in South Asian (27.3%) and Arabian Peninsula (12.8%) populations for *APC* and some Asian populations (26.5%) for *KRAS* [24]. Colorectum cancer is likely to require constitutive Wnt activation through mutation with inactivation of BMP/TGFβ signaling and Ras pathway activation, and it has recurrently mutated non-coding elements including *APC* and *SMAD4* splice regions and *ST6GALNAC1* distal promoter [25]. 

#### 2.1.2. Gastric Cancer

Gastric cancer is the fifth type of cancer with the highest incidence after breast, lung, colorectum, and prostate, and it is the third leading cause of cancer-associated deaths after lung and colorectum worldwide [9]. Less than 3% of gastric cancer patients may have a hereditary form, and tools for assessing genetic risk are limited. Certain germline genetic syndromes are associated with inherited susceptibility, including hereditary diffuse gastric cancer syndrome (*CDH1*—cancer risk 70%), Peutz–Jeghers syndrome (*STK11*—cancer risk 29%), juvenile polyposis syndrome (*SMAD4*—cancer risk 21%), Lynch syndrome (*MLH1*, *MSH2*, (*EPCAM* deletions), *MSH6*, and *PMS2*—cancer risk 1–13%), Li–Fraumeni syndrome (*TP53*—cancer risk 1–4%), familial adenomatous polyposis (*APC*—cancer risk < 1%), and a variant of FAP gastric adenocarcinoma and proximal polyposis of the stomach (APC promoter 1B—cancer risk significantly elevated) [26]. Gastric cancer also has been reported in families with other homologous recombination DNA-repair pathway syndromes related to pathogenic variants in *BRCA2* and *PALB2* [27]. 

Molecular subtypes of gastric cancer classified via genomics include (1) chromosomal instability (49.8%), which most corresponds to the intestinal type and has *TP53* mutations and tyrosine kinase receptor–RAS signal amplification; (2) genome stability (19.7%), which most corresponds to the diffuse type according to the histopathological classification and has *CDH1* and *RHOA* mutations and *CLDN18-ARHGAP* fusion inactivated cell adhesion; (3) MSI (21.7%), which has high tumor mutation burden and DNA-hypermethylation-activated mitosis; and (4) EBV positivity (8.8%), which corresponds to DNA-methylations-activated immune systems. In MSI and EBV positivity, *PIK3CA* and *ARID1A* gene mutations are enriched and in EBV-positive subgroups; PD-L1/2 is often overexpressed via gene amplification and structural variation [28]. Chemotherapy and molecularly targeted therapies are now accompanied by other therapeutic agents like immune checkpoint inhibitors (ICIs) selected for the appropriate patient, as humanized monoclonal antibodies that target inhibitory receptors like CTLA-4, PD-1, LAG-3, TIM-3, and PD-L1 expressed on T lymphocytes, antigen-presenting cells, and tumor cells elicit an anti-tumor response by stimulating the immune system [29]. In the MSI subtype, ICIs are effective due to the presence of high-frequency gene mutations and neoantigen expression, while in the genome stability subtype, ICIs are quite ineffective (12%), as there are a few genomic abnormalities, including chromosome amplification and deletion, compared with subtypes MSI and EBV positivity, with 85.7 and 100.0% efficacy [30]. 

#### 2.1.3. Lung Cancer

Lung cancer is the second type of cancer with the highest incidence after breast, and it is the first leading cause of cancer-associated deaths worldwide [9]. A large number of somatic and germinal mutations (*EGFR* (20%), *TP53* (54.6–64.6%), *KRAS* (43.7%), *BRAF* (3.2%), *ERBB2* (1.3%), *MET* (9.4%), *STK11* (16.2%), and *PIK3CA* (9−12.4%)), gene amplifications (*EGFR*, *ERBB2*, *MET* (17.68%), *PIK3CA*, and *NKX2*), deletions (*DOK2*), rearrangements (*ALK* (13.3%), *ROS1* (3.9%), and *RET* (5.2%)), and fusions (*ALK/EML4*) [31], which increase the risk of developing lung cancer in certain populations, have been identified but have not led to the development of effective treatments since global mortality rates have not significantly decreased [9]. *TP53* (78%), *TTN* (68%), *CSMD3* (39%), *MUT16* (36%), and *RYR2* (36%) have the highest mutational frequency, and *BRINP3*, *COL11A1*, *GRIN2B*, *MUC5B*, *NLRP3*, and *TENM3* have shown significantly higher mutational frequency in stage III of lung squamous cell carcinoma [32], while *TP53*, *EGFR*, *KRAS*, *ALK*, *BRAF*, *MET*, *RET*, and *ROS1* are the most frequent mutation genes in lung adenocarcinoma [33]. *KRAS* mutations appear in less than 40% of NSCLC patients, with a higher prevalence in LUAD (32%) than in LUSC (4%), in Western (26%) than in Asian (11%) patients, in smokers (30%) than in non-smokers (10%), and almost never in SCLC [34]. EGFR mutation frequency is much higher in NSCLC never-smokers (49.3%) than in smokers (21.5%), in females (43.7%) than in males (24.0%), and in LUAD (38.0%) and Asians (38.4%) [35]. 

The mutational status of *EGFR*, *KRAS*, and *TP53* has been used to classify lung adenocarcinoma patients into seven subtypes that show a relationship with prognosis with available survival data, and the associations between classification and clinicopathologic variables, including demographic characteristics, smoking history, fluorescence in situ hybridization, and molecular results, have found better overall survival in patients who underwent surgery and had tumors enriched for *EGFR* mutations, which are nearly exclusively found in non-small cell lung carcinomas, and worse overall survival with older age, stage IV disease, and tumors with co-mutations in *KRAS* and *TP53* [36].

### 2.2. Transcriptomics Studies

The bioinformatic analysis of high-throughput sequencing methodologies such as microarrays and RNA sequencing allows to identify all the transcriptionally deregulated genes involved in the modulation of biological processes and signaling pathways related to tumor cell grade, differentiation status, metastatic potential, and patients’ survival [37,38]. The human genome has information for around 1400 regulatory genes known as transcription factors (TFs), representing about 6% of all human protein coding genes. DNA-binding transcription factors recognize cis-regulatory elements of target genes, which make them the most direct regulators of gene transcription during cellular differentiation, development, and response to external factors through the activation and/or inhibition of specific signaling pathways [39]. In normal and tumor cells, TFs coding genes can be regulated positively or negatively by genetic and epigenetic mechanisms related to transcriptional expression, like point mutations and DNA methylation; as well as post-translational modifications that regulate their functional state, like phosphorylation, acetylation, SUMOylation, and ubiquitylation; and mechanisms that include control over protein localization or binding site, all of which result in a loss or gain of function [40,41]. 

#### 2.2.1. Colorectum Cancer

In colorectum cancer, three transcriptomic analyses have compared tumor cases and healthy controls [42,43]. The first identified the differences in global transcriptional regulatory programs of normal and tumor colon cells, showing a large reduction of transcriptional interactions in the tumor network, TFs, and target genes, while the average gene expression was conserved, and 91 TFs increased their connectivity in the tumor network [42]. Another colorectum cancer study analyzed the signatures of co-deregulated genes and their transcriptional regulators in five datasets, highlighting 17 hub-co-upregulated genes and 18 hub-co-downregulated genes including three well-known TFs as well as three kinases as critical genes [43]. Another study used four microarray datasets and one RNA-seq dataset identifying 50 common DEGs, of which 11 were identified as key genes, significantly associated with colorectum cancer progression along with four TFs and eight microRNAs as their key transcriptional and post-transcriptional regulators [44]. 

#### 2.2.2. Gastric Cancer

In gastric cancer, three transcriptomic analyses have compared tumor cases and healthy controls. The first analyzed only one microarray dataset and made a protein–protein interaction network with the Retrieval of Interacting Genes database, identifying the top five TFs according to the calculation of the regulatory impact factor [45]. Another study analyzed one microarray dataset and TCGA-STAD datasets, identifying 222 overlapping genes, from which 4 demonstrated an important statistical correlation with the diagnosis and prognosis of gastric cancer [46]. Recently, an algorithm named mRBioM was developed for the identification of 55 potential mRNA biomarkers from complete transcriptomic RNA profiles of 279 patients with gastric adenocarcinoma [47], of which some were TFs and cell receptors, but the study did not identify the main regulators of transcription related to acquisition of the hallmarks of cancer or suggest any other biological process that might be involved during tumorigenic process. 

#### 2.2.3. Lung Cancer

In our lung cancer bioinformatic analysis of TF RUNX2 [48], we analyzed and compared our pipeline and results [38,49] with previous studies, finding eleven publications assessing the lung cancer TRN with different microarray and RNA-seq studies; we also performed direct and very different bioinformatics analyses on datasets created or selected from the databases. Four studies were led with cell lines; five were made with one, two, or six microarray studies; and two were conducted with TCGA RNA-seq studies. None attempted to perform a global analysis of lung cancer, and most conducted the analysis with a reduced number of datasets for each subtype of lung cancer independently. None of them tried to select deregulated genes unique to lung cancers that are not deregulated in other lung diseases or other types of cancer, and none performed a joint coregulatory analysis to study the cooperative and coordinated regulatory functions of TFs. However, the fourteen TFs identified using our bioinformatics pipelines are also in some of the other studies, but their regulatory functions during tumor processes were not properly analyzed. Therefore, regardless of the cell types, the detection methodology of gene expression, and the bioinformatics methodology used, there is a group of regulatory genes or TFs (*SOX4*, *SOX17*, *BZW2*, *FOXM1*, *ZBTB16*, *TAL1*, *KLF4*, *EPAS1*, *HOXC6*, *ID4*, *KLF2*, *MEIS1*, *NR2F1*, *TBX4*, *TCF21*, *TFAP2C*, *LMO2*, *MNDA*, *FOXF1*, *HLF*, *RFX2*, *DLX5*, *MYBL2*, *NR4A3*, *PKNOX2*, and *GPRASP1*) that are consistently co-expressed in a statistically significant manner in lung cancer [37]. 

In our lung cancer bioinformatic analysis, we also analyzed the hypothesis of establishment or “quasi-malignancy”, which suggests that lung cancer and severe pulmonary arterial hypertension (PAH) share several hallmarks of cancer [50]. In PAH, many cells in the vascular wall become abnormal trying to survive under stress conditions such as inflammation and pseudo-hypoxia, mainly showing phenotypic, angiogenic, and glycolytic switches like tumor cells, which are related to the reduction of oxygen consumption, mitochondrial respiration, the increase glycolytic metabolism [51], genome instability, mutations, inflammation, avoidance of immune destruction, and the ability to evade apoptosis [52]. PAH showed great complexity comparable to lung cancer, evidenced by the significant number of differentially expressed genes (DEGs), like those observed in lung cancer, which might be related to the difficulty in treating PAH [38]. Moreover, our pipeline identified a lung cancer TRN of twenty-six TFs, of which nineteen are also deregulated in PAH and co-expressed in gene networks as regulators of the most frequently dysregulated DEG in the two types of lung cancer (non-small-cell lung cancer and small-cell lung cancer) and PAH. Furthermore, several TFs from the network showed experimental evidence related to the acquisition of cancer stem-like characteristics and differentiation to cancer cells. Our results indicate that lung cancer has unique and common deregulated genes and TFs with PAH, co-expressed and regulated in a coordinated and cooperative manner by the transcriptional regulatory network that might be associated with critical biological processes and signaling pathways related to the acquisition of the hallmarks of cancer, making them potentially relevant tumor biomarkers for lung cancer early diagnosis and targets for the development of personalized therapies against lung cancer [37].

### 2.3. Epigenomics Studies

Different methylation patterns have been related to every cancer type, the mutational status of cancer driver genes, and various epidemiological and environmental factors [53]. DNA methylation is the covalent addition of a methyl group (-CH3) to the position five of a cytosine (5-methylcytosine (5mC)), mainly in cytosine–guanine (CpG) dinucleotides catalyzed by the DNA methyltransferases, including DNMT1, DNMT3A, and DNMT3B, with S-adenosylmethionine (SAM) being the donor of the methyl group and the ten-eleven translocation (TET) family the indirect mediator of DNA demethylation through the oxidization of 5mC. CpG methylation is a known a repressive mark associated with long-term gene silencing occurring in ~80% of all CpG sites containing, nonetheless, a few CpGs form clusters or CpG islands (CGIs) related to regulatory regions, which are normally hypomethylated and have transcription-factor-binding sites [54]. Therefore, DNA methylation in the promoter region may regulate the binding of methylation-sensitive TFs and consequently the expression of target genes [55]. DNA methylation might result in the disruption of genome topology, driving aberrant regulatory interactions and abnormal gene expression in cancer [56]. The cancer transcriptional dysregulation arises from disease-defining indirect genetic alterations, via mutation of signaling factors, or directly via genetic alterations in gene regulatory factors, affecting proteins participating in almost all levels of transcriptional control, including trans-factors (TFs, signaling proteins, cofactors, chromatin regulators, and chromosome-structuring proteins) and cis-elements (enhancers, promoters, and insulators) [57]. 

#### 2.3.1. Colorectum Cancer

The current molecular pathogenesis of colorectum cancer includes chromosomal instability, MSI, epigenetic instability such as CpG island methylator phenotype (CIMP), altered tumor microenvironments, and metabolic state [20]. Genome-wide methylation sequencing has shown global hypomethylation, which is associated with increased genomic instability, and CGIs hypermethylation of primary colorectum tumors. Gene ontology analysis has revealed shared biological processes between hypermethylated CGIs in metastasis and primary tumors. Five genes (*FIGN*, *HTRA3*, *BDNF*, *HCN4*, and *STAC2*) have the potential to become methylation biomarkers of poor prognosis in colorectal cancer patients, according to the Cancer Genome Atlas (TCGA) cohort analysis that associated them with poor survival [58]. Changes in DNA methylation have been suggested as useful new biomarker candidates depending on the sample source, namely plasma, stool, urine, and surgically removed tumor tissues, used for diagnosis and for the determination of prognosis and treatment response [59]. The prognosis of patients with high circulating tumor DNA (ctDNA)-methylation levels was proven to be worse, indicating its great value in monitoring colorectum cancer relapse [60]. Hypermethylation of cell-free DNA from the blood or stool is considered as a potential non-invasive colorectum cancer biomarker, suggesting several methylation markers that have shown high sensitivity and specificity for screening and early detection (*SEPT9*, *SDC2*, *MGMT*, *NDGR4*, *APC*, *BMP3*, and *VIM*), prognosis (*SFRP*, *p16*, *LINE-1*, *BCAT1/IKZF1*, and *RASSF1A*), and prediction for response to treatment (*hMLH1*, and *Wnt5A*) [61]. Mutations in *BRAF* and *KRAS* genes affect different biological pathways and are functionally able to dysregulate DNA methylation [62] and miRNA expression [63]. The methylation profile of tumoral tissues with the BRAF V600E mutation has shown increased methylation levels of *SFRP2*, *DKK2*, *PCDH10*, *TMEFF2*, *SFRP1*, and *HS3ST2* compared with tissues without this mutation [22]. 

#### 2.3.2. Gastric Cancer

Molecular aberrations like aberrant chromatin structures, gene mutations, structural variants, and somatic copy number alterations are involved in gastric tumorigenesis. The presence of multiple DNA-methylation patterns in gastric cancer have allowed their classification into distinct molecular subgroups: the extremely high-methylation epigenotype uniquely related to the EBV-positive subtype, high-methylation epigenotype associated with MSI, and low-methylation epigenotype. The EBV-positive subtype also shows *CDKN2A* silencing, *PIK3CA* mutations, *PD-L1/2* overexpression, and lack of *TP53* mutations. The MSI subtype often has *MLH1* silencing and abundant gene mutations. The genome stability subtype is generally a diffuse type that frequently shows *CDH1/RHOA* mutations or *CLDN18–ARHGAP* fusion. Chromosomal instability is generally an intestinal type that frequently has *TP53* mutations and genomic amplification of receptor tyrosine kinases [64]. Pathogens invade host cells and cause epigenetic changes such as DNA methylation, making it a safer environment for the pathogen and allowing the infection to endure and promote gastric cancer [65]. *Helicobacter pylori* infection induces hypermethylation in the promoter regions of DNA-repair genes and tumor-suppressor genes *RPRM* and *ZNF793* [66] as well as *MHL1*, *RUNX3*, *APC*, and *PTEN*, generating silencing and tumorigenesis [67]. Gastric cancer can be divided into three epigenotypes according to DNA-methylation patterns and EBV infection: EBV(−)/low methylation, EBV(−)/high methylation, and EBV(+)/high methylation. There are genes methylated only in the EBV(+) tumors (*CXXC4* and *TIMP2*), genes methylated in both EBV(+) and EBV(−)/high tumors (*COL9A2*, *EYA1*, and *ZNF365*), and genes methylated in all gastric cancers (*AMPH*, *SORCS3*, and *AJAP1*) [68]. The gene expression level of TGFβ2 changes in different tumor stages, T categories, grades, and patients’ survival states is upregulated in gastric cancer patients, and its expression could be affected by the methylation site cg11976166 located on its promoter [69]. The regulatory mechanisms and functional roles of all deregulated genes of the DNA-methylation signature must be studied to understand the role in tumorigenesis and the development of diagnostic and treatment strategies against gastric cancer. 

#### 2.3.3. Lung Cancer

Epigenetic changes including DNA methylation, histone modification, non-coding RNA expression, and DNA methylation have also been reported in lung cancer. Gene methylation levels of *SHOX2* and *RASSF1A* display higher specificity and sensitivity for early lung cancer detection than the traditional cytological method as well as dual genes (*RASSF1A-RARβ2*, *SHOX2-PTGER4*, and *p16-RARβ2*, which could become possible biomarkers for early diagnosis [70]. Increased levels of methylation of *DAL-1*, *EPHB6*, *HS3ST2*, *TMEM88*, and *MGMT* and decreased levels methylation of *ELMO3* are markers specifically associated with lung cancer progression and higher rates of metastasis. The methylation levels of *APC*, *HOXA9*, *RARβ2*, and *RASSF1A* are distinctive of lung cancer subtypes and stage [71]. *APC*, *CDH13*, and *CDKN2A/P16* are more frequently hypermethylated in NSCLC, while genes related to apoptosis pathways (*CASP8*, *TNFRSF6/Fas*, and *TRAIL-R1/DR4*) are frequently methylated in SCLC cell lines and tumors [71]. *HOXA9* and *RASSF1A* have higher methylation levels in SCLC than NSCLC in cfDNA [72]. The methylation rates of *ANK1*, *APC*, *CCND2*, *CDH13*, *GATA3*, *KCNH5*, *LINE-1*, *RARβ*, *RASSF1*, and *RUNX3* are significantly higher in LUAD. Six genes (*CLDN1*, *TP63*, *TPX5*, *TCF21*, *ADHFE1*, and *HNF1B*) were identified as potential DNA-methylation markers for LUSC diagnosis compared to a non-tumor lung [73]. *KRAS* mutations in isogenic lung cancer cell lines have differentially methylated CpGs and an enrichment for genes involved in development and differentiation [74]. *EGFR* is significantly hypomethylated in LUAD tumors of TCGA, and *EGFR* CpGs in the promoter region is negatively correlated with the transcription level, protein expression, and somatic copy number variation, while the methylation at the gene body region is positively correlated with these features [75]. *TP53* mutations might be able to affect global DNA methylation through DNMT1 overexpression in lung cancer and increased genomic instability [71].

Recent studies have observed congruent genomic, transcriptomic, and DNA methylation evolutionary trajectories in several types of cancers. At the transcriptional level, the role of the dysregulation of DNMTs, TETs, and other related proteins like Polycomb protein EZH2 can be studied in order to analyze the importance of changes in DNA-methylation patterns in cancer establishment and progression and how the dynamic remodeling of DNA methylation is essential for the development and cell-fate decisions as cancer stem cells exit pluripotency during their differentiation [71]. Studies integrating the genomic, epigenomic, and transcriptomic profiles of cancer all in the same set of individuals that represent a certain population are essential to better understand tumor evolution, systematically characterize the genomic and epigenomic landscape according to the TRN function and analyze the molecular impact of the microbiome network in a tumor disease. The multiomics study of oral-gut-lung axis provides an outstanding opportunity to identify regulatory proteins as key biomarkers related to the acquisition of the hallmarks of cancer for the future development of targeted epidrugs for treatment of complex tumorigenic diseases. Nonetheless, as can be seen in this section, the results of omics studies are being analyzed in a traditional way, looking for specific biomarkers without arriving at a more holistic analysis capable of making a full correlation between the results of the different omics techniques.

## 3. Microbiome Implicated in the Oral-Gut-Lung Axis

**Figure 1:**
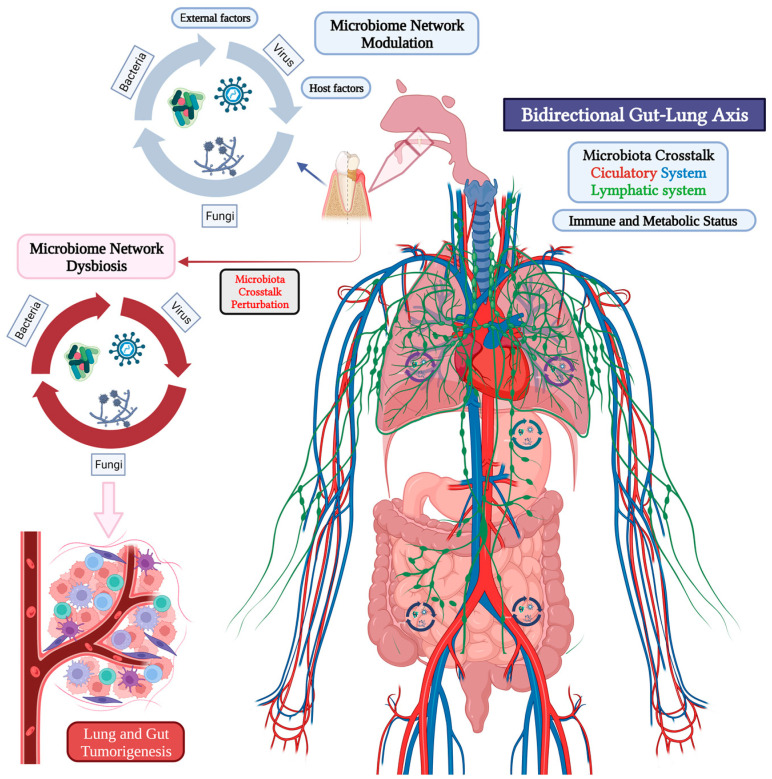
Microbiome crosstalk in the oral-gut-lung axis. Lymphatic system (Green), Circulatory system (Arteries in red and Veins in blue). Created with BioRender.com.

Microbiomes colonize different environments, including living organisms, and influence the health of the niche that they inhabit. Methodologies based on high-throughput sequencing data of biological samples produces taxonomical profiles (metagenomics), functional profiles (metatranscriptomics), and metabolic profiles (metabolomics) that cover the byproducts released into the environment. Each approach provides valuable information separately but when combined in bioinformatic network-based methods can be applied to integrative studies, which might hold the key to in-depth understanding of microbiomes [76]. The human microbial community is composed by millions of microbes expressing as many as or even more genes than the host, so it has been defined as a complex, vital organ forming a multidirectional connecting axis with host cells from other organs, allowing the communication of neural, endocrinal, humoral, immunological, and metabolic pathways and usually associated with the host’s immunity and ability to defend against pathogenic invasion [77]. The dysbiosis of the microbiota must therefore be linked to the mechanism leading to the development various complex human diseases such as cancer, as many recent worldwide clinical studies are demonstrating the relationship between specific microbial species dysbiosis and disease as well as eubiosis in health, endorsing their benefits as probiotics and their ability to aid in the treatment of various infectious diseases, dysfunctions of the gastrointestinal tract, and inflammatory disorders [78]. The oral-gut-lung axis is a bi-directional communication network connecting oral, gastric, intestinal, and pulmonary microbiota and is considered responsible for the massively increased microbial load, causing alterations in oral, airway, and gut microbiota and their transitory translocation into the lymphatic system and circulatory system toward the bowel (Figure 1) [5]. Currently, metagenomic studies are the initial phase for identifying all the microorganisms related to the oral cavity and gastrointestinal and respiratory systems and starting to acquire a comprehensive understanding of microbiota interaction and its role in health, inflammatory, and tumorigenic processes.

### 3.1. Oral Microbiota

The human microbiome project has revealed the vast diversity of the oral microbiome, with several studies reporting around 700 species in the oral cavity and 500 within the subgingival biofilm [78]. The microorganisms are present in saliva, gingival epithelium, and internal surfaces of the oral cavity, but they are concentrated in dental plaque. The oral cavity contains multiple genera of anaerobic bacteria, such as *Actinomyces*, *Arachnia*, *Bacteroides*, *Bifidobacterium*, *Eubacterium*, *Fusobacterium*, *Lactobacillus*, *Leptotrichia*, *Peptococcus*, *Peptostreptococcus*, *Propionibacterium*, *Selenomonas*, *Treponema*, and *Veillonella* [79], and fungi such as *Candida*, *Cladosporium*, *Aspergillus*, *Fusarium*, *Glomus*, *Alternaria*, *Penicillium*, and *Cryptococcus* [80]. Oral mycoses are uncommon, but when they appear, they cause great discomfort and sometimes the destruction of tissues. Immunodeficiency viruses and immunosuppressive drugs have been related to the increase in the frequency of fungal infections, as most are an outcome of opportunistic conditions when immune status is compromised. The most common fungal infection of the oral cavity is candidiasis; however, it can be a part of normal commensals. *Candida albicans* is the most frequent type, but oral lesions are also produced by *Candida parapsilosis*, *Candida krusei*, *Candida stellatoidea*, *Candida tropicalis*, *Candida glabrata*, *Candida guilliermondii*, *Candida dubliniensis*, and *Candida auris*. Cytology and tissue biopsy can confirm the clinical diagnosis, and the treatment is focused on signs, symptoms, and culture reports [81]. Subgingival plaque from periodontitis has been associated with genera such as *Filifactor*, *Treponema*, *Porphyromonas*, *Tannerella*, *Eubacterium*, *Peptostreptococcaceae*, *Desulfobulbus*, *Lachnospiraceae*, *Alloprevotella*, *Hallella*, *Mogibacterium*, *Phocaeicola*, *Johnsonella*, and *Mycoplasma* [82]. Viral infection has been associated with herpes simplex virus (HSV), Epstein–Barr virus (EBV), and human cytomegalovirus (HCMV) and has been also linked to the development and progression of periodontal diseases [83]. The oral cavity is particularly susceptible to viral infections in soft tissue and salivary glands, including primary lesions formed by HSV and human papillomavirus (HPV) and secondary pathological processes of bacterial or fungal nature due to viral immunosuppression, such as the human immunodeficiency virus (HIV) [84]. 

The oral microbiome is translocated to the intestine initially through the gastrointestinal tract and subsequently through the systemic circulation, including pathogenic microorganisms that can alter intestinal homeostasis, such as periodontal pathobionts, which promote the disordered state of the intestinal microbiota, triggering an immunity response [85]. Sequencing-based studies have identified an intestinal enrichment of oral-associated bacteria with the ability to induce intestinal inflammation in mice models, suggesting an oral cavity origin of the intestinal pathobionts, particularly members of the genus *Streptococcus*, found in saliva and fecal samples of inflammatory bowel disease (IBD) patients [86]. Moreover, oral bacteria have shown more complexity in patients with gastric and colorectal cancer compared with the normal control population, like dysbiosis of *Fusobacterium nucleatum* and *Porphyromonas gingivalis* [87] as well as *Neisseria mucosa*, *Prevotella pleuritidis*, *Mycoplasma orale*, and *Eubacterium yurii* [88]. 

The oral microbiota is the main source of the lung microbiome because when breathing with the mouth, a person can inhale saliva into lower respiratory tract, and when a person coughs, this could make the mucus and substances of the respiratory tract enter the mouth [89]. Several oral pathogens like *A. actinomycetemcomitans*, *Actinomyces israelii*, *Capnocytophaga* spp., *Chlamydia pneumoniae*, *Eikenella corrodens*, *F. nucleatum*, *Fusobacterium necrophorum*, *P. gingivalis*, *P. intermedia*, and *Streptococcus constellatu* have been implicated in lung diseases like pneumonia and respiratory tract infections [90]. The relative abundances of oral *Burkholderia*, *Lautropia*, and *Ralstonia* have been shown to correlate with PAH [91,92]. Moreover, a higher relative abundance of *Abiotrophia*, *Lactobacillus*, and *Streptococcus* and detectable *Peptoniphilus* in the oral cavity have been associated with a greater risk of developing lung cancer, particularly squamous cell carcinoma among former smokers, whereas individuals with detectable *Peptostreptococcus*, *Eubacterium yurii*, or *Aggregatibacter* had a lower risk of developing lung cancer. Most of these genera have also been associated with pneumonia, periodontal disease, or dental caries and decay as well as lung infections, periodontal disease, and lung cancer risk [93]. 

### 3.2. Gut Microbiota

The five main bacteria phyla of the gastrointestinal tract are Firmicutes (60–80%) (*Clostridium*, *Ruminococcus*, *Lactobacillus*, and *Enterococcus*), Bacteroidetes (15–25%) (*Bacteroides*, *Prevoetella*, and *Xlanibacter*), Actinobacteria (2.5–5%) (*Bifidobacterium*), Proteobacteria (1–10%) (*Escherichia* and *Enterobacteriaceae*), and Verrucomicrobia (0.1–2.2%) (*Akkermansia muciniphila*) [94]. The colonization of the gastrointestinal tract begins at vaginal birth with *Bacteroides* and *Bifidobacteria*; breastfed infants are colonized with *Bifidobacterium* and *Lactobacillus* and formula-fed infants with *Enterococcus*, *Enterobacteria*, *Bacteroides*, *Clostridium*, and *Streptococcus* bacteria, and the microbiome composition and diversity change throughout a person’s life [95]. The stomach and duodenum have a very low bacterial species concentration, which is just around one hundred, while the colon has over two hundred, specifically in the cecum and right colon due to the favorable pH, substrates, and nutrients available, which creates an advantageous setting for bacterial growth [96]. There is a bi-directional relationship among nutrients provided by Western-, Eastern-, European-, American-style diets and gut microbiota, where diet components and patterns in every population and every individual can modulate gut microbiota composition and contribute to host physiology and metabolism, while gut microbiota can metabolize dietary components into several microbial-derived metabolites and diet components [97]. A nutritionally balanced and fiber-rich diet provides all necessary nutrients and nourishes a healthy gut microbiome with a high diversity and well-balanced composition, although many modern food-processing ingredients have harmful effects on the intestinal barrier, reducing gut microbiota diversity and composition, promoting obesity, and possibly predisposing to intestinal inflammation in susceptible subjects [98]. 

Long-standing IBD, either Crohn’s or ulcerative colitis, increases the risk of overall cancer and cancer-specific mortality, particularly digestive cancers [99]. IBD has been associated with *Clostridium difficile*, *Mycobacterium avium* subspecies *paratuberculosis*, enterotoxigenic *Bacteroides fragilis*, *Fusobacterium varium*, adherent-invasive *Escherichia coli*, enteric *Helicobacter* species, *Campylobacter concisus*, and *Fusobacterium nucleatum*. Likewise, a pathogenesis model has been proposed that illustrates the role of the intestinal initiator and commensal bacterial species in the development of IBD [100]. In the early stage of IBD development, the initiating enteric pathogens (*C. concisus*, *F. nucleatum*, *B. fragilis*, *F. varium*, adherent-invasive *E. coli*, and *C. difficile*) with different virulence factors induce inflammation with help from proinflammatory cytokines, overcome the mucosal defense system, and damage the intestinal epithelial cells. In the second stage, there is a continuous supply of *C. concisus* and *F. nucleatum* to the intestinal tract from the oral cavity, and resident enteric microbes increase long-term passage of intestinal commensal microbes and their products into intestinal tissues and promote mucosal immune system responses to commensal bacteria, breaking down the homeostasis between the mucosal immune system and the gut microbiota. In the third stage, intestinal commensal microbes become the main drivers of intestinal inflammation, due to their large numbers, through innate and adaptive immune responses [100]. Therefore, when a local dysbiosis occurs, both initially at the oral level and later at the intestinal level or vice versa, multiple microorganisms together with inflammatory cytokines and other metabolites can spread to other parts of the organism through the gastrointestinal tract and blood circulation in a cyclical manner, where they could trigger or aggravate other tumor diseases of inflammatory origin [101].

The gut microbiota may promote tumorigenesis by inducing oxidative stress, genotoxicity, host immune response disturbance, and chronic inflammation [1]. Colorectal and gastric cancer are the major gastrointestinal tract cancers around the world, with East Asia, Eastern Europe, and South America being the hotspots for incidence and mortality, where rates vary depending on geographic location, host genetics, and the evolutionary lineage of the microbiota [102]. The dominant bacterial species in colorectum tumorigenic evolution are still unknown; nonetheless, preclinical and clinical sample-based studies support a possible role in susceptibility or progression, with higher proportions of *Fusobacterium nucleatum*, *Bacteroides fragilis*, *Escherichia coli*, *Enterococcus*, *Campylobacter*, *Peptostreptococcus*, *Shigella*, *klebsiella*, and *Akkermansia* and lower levels of *Ruminococcus*, *Bifidobacterium*, *Eubacteria*, and *Lachnospira* compared with healthy subjects [103,104]. *Helicobacter pylori* has been the best-known microbial infection related to cancer in the human gastrointestinal tract since early 1990s [105]. Several studies have shown the relationship between these flagellated Gram(−) bacteria and the onset of gastric adenocarcinoma by increasing gastric pH, creating favorable niches for bacterial colonization, and changing the gastric microbiota composition [102]. Vacuolating cytotoxin A is an intracellular-acting and channel-forming toxin of *Helicobacter pylori* that impairs host endolysosomal trafficking, induces the accumulation of dysfunctional lysosomes and autophagosomes, and increases reactive oxygen species, mitochondrial damage, and inflammation, thus attenuating the host immune response and facilitating its own colonization in the stomach [106]. Cytotoxin-associated gene A is a *Helicobacter pylori* strain-specific protein transferred into host cells by the type IV secretion system to inhibit the apoptotic pathway, cause loss of cell polarity and adhesion, and increase cell motility, scattering, and elongation of epithelial cells [107]. Although *Helicobacter pylori* might induce chronic gastritis and peptic and duodenal ulcers and is linked to more than 90% of gastric cancer cases, only 1 to 3% of infected individuals progress to gastric cancer [108]. In Colombia, the prevalence of *Helicobacter pylori* is 90% [109], the mortality of gastric cancer is the highest in men, and the incidence varies geographically [9]. Infection by *Helicobacter pylori* is prevalent in populations in the mountains and the coast, and specifically in Nariño, there is a very high incidence and mortality of gastric cancer, as in Eastern Asian populations; however, in Tumaco, where the *Helicobacter pylori* infection is nearly universal, the incidence and mortality rates for gastric cancer are quite low, as in African populations [109]. Until now this “Colombian Enigma” has not had an appropriate explanation, but it might be related to the absence of the microbiome network that accompanies *Helicobacter pylori* and is essential for generating the specific group and sequence of signals that initiate the tumorigenic process. Metagenomics have identified other acid-resistant bacteria in the stomach of gastric cancer patients besides *Helicobacter pylori* at the phylum level, namely *Firmicutes*, *Bacteroidetes*, *Actinobacteria*, *Fusobacteria*, and *Spirochetes*, and at genus level, namely *Lactobacillus, Streptococcus*, *Faecalibacterium*, *Bacteroides*, *Acinetobacter*, *Prevotella*, *Sphingomonas*, *Fusobacterium*, *Comamonas*, and *Empedobacter* [110]. 

The human gastrointestinal tract is also home to many fungi, representing 0.1% of the total gut microbes, they play crucial roles in human intestinal immune homoeostasis and disease pathogenesis and contribute to health and disease. The fungal genera in the gastrointestinal tract include *Candida*, *Saccharomyces*, *Aspergillus*, *Penicillium*, *Rhodotorula*, *Trametes*, *Pleospora*, *Sclerotinia*, *Galactomyces*, and *Bullera* [111]. In newborns, gut fungi are determined by maternal diet, delivery mode, gestational age at birth, infant feeding mode, and environment and dominated by *Saccharomycetales* and *Malasseziales* spp. In adulthood, population geography, ethnicity, urbanization, lifestyles, and dietary habit remain the major determinants in configuring gut mycobiota composition, which is dominated by the phyla *Ascomycota*, *Basidiomycota*, and *Zygomycota*. The healthy adult gut mycobiome is mainly composed of *Candida*, *Saccharomyces*, and *Cladosporium*. In Crohn’s disease, the fecal fungal load increases in the *Basidiomycota*-to-*Ascomycota* ratio, *Candida albicans*, *Candida tropicalis*, *Candida glabrata*, *Aspergillus clavatus*, *Cryptococcus neoformans*, and *Debaryomyces hansenii*, while *Saccharomyces cerevisiae* decreases. In ulcerative colitis, *Debaryomyces* and *Candida albicans* increase, while *Alternaria alternata*, *Aspergillus flavus*, *Aspergillus cibarius*, and *Candida sojae* decrease. In colorectal cancer, the *Basidiomycota*-to-*Ascomycota* ratio, *Trichosporon*, and *Malassezia* increase, while alpha diversity decreases. In gastric cancer, *Candida* and *Alternaria* increase, while *Saitozyma* and *Thermomyces* decrease [112]. 

Eukaryotic viruses include rotavirus, astrovirus, calicivirus, norovirus, hepatitis E virus, coronavirus, torovirus, adenovirus, *Picornaviridae*, *Reoviridae*, the Enterovirus genus, plant-derived viruses, giant viruses that have been detected in the intestine along with prophages, and the Microviridae family (*Microvirus*, *Gokushovirinae*, *Alpavirinae*, and *Pichovirinae*) [113,114]. The gut virome in neonates is composed of phages from the Caudiovirales order (*Myoviridae*, *Siphoviridae*, and *Podoviridae* family), which infect the pioneer bacteria, followed by eukaryotic virus diversification associated with environmental exposures, particularly breastmilk [115]. Healthy adult stool is richer in *Podoviridae*, *Siphoviridae*, *Myoviridae*, and *Microviridae* families as well as *Virgaviridae* viruses of plant origin, suggesting dietary influence in the composition [116]. *Hepadnaviridae* is highly abundant along with the small protein HBx, while *Polydnaviridae* and *Tymoviridae* are less abundant in ulcerative colitis. Hepatitis E virus (HEV)-derived proteins in Crohn’s disease patients may also have an impact on host immunity, eventually triggering intestinal inflammation, while *Virgaviridae* is less abundant [117]. Oncoviral infection is responsible for around 15% of human cancer, and around 10% of gastric adenocarcinomas are EBV-positive, so early-stage clonal viral integration of EBV can be identified by performing virome-wide screening, evidencing the likely participation of these viruses in early stages of tumorigenesis [118]. Enrichment of inovirus and tunalikevirus may represent a new paradigm of trans-kingdom microbial interaction mediated by the colorectal cancer virome because they are able to infect Gram-negative bacterial hosts like bft-positive enterotoxigenic *Bacteroides fragilis* and *Fusobacterium nucleatum* and pks-positive genotoxic *Escherichia coli*, which are implicated in colorectal development, probably through horizontal and vertical transmission of virulence genes encoded by a filamentous bacteriophage that might be related to the evolution of bacterial virulence within the natural biofilm microbiome. The gut-oral bacterial species might also have trans-kingdom microbial interactions with bacteriophages in a colorectal tumor-node-metastasis stage–dependent manner, playing a specific regulatory role in the survival of microbiome Streptococcus species [119].

### 3.3. Lung Microbiota

The lungs were previously thought to be sterile, but now, they are known to harbor dominant phyla in the upper respiratory tract (2.2 × 10^3^ bacterial genomes per cm^2^), including *Bacteroidetes*, *Firmicutes*, *Proteobacteria*, *Actinobacteria*, *Fusobacteria*, and *Saccharibacteria*, which partially overlap with the intestinal microbiota [91]. Microbiota constitution is controlled by immigration and elimination rates of the microorganisms in the healthy lung and by the regional growth conditions that affect replication rates in the diseased lung [120]. The dynamic microbial system is often theorized as a pendulum that swings between two states, health and disease, from a paucity of microbes in the lung airways to patterns of resilient microbial colonization [121]. The human core gut and lung microbiota are similar at the phylum structure level but differ in their bacterial species composition, with *Haemophilus* spp., *Pseudomonas* spp., *Streptococcus* spp., and *Veillonella* spp. frequently found in the airways. The dominant taxa in the nasal cavity and nasopharynx include *Moraxella*, *Staphylococcus*, *Corynebacterium*, *Haemophilus*, and *Streptococcus* species, whereas the oropharynx exhibits high abundance of *Prevotella*, *Veillonella*, *Streptococcus*, *Leptotrichia*, *Rothia*, *Neisseria*, and *Haemophilus* species; the lower respiratory tract, trachea, and lungs exhibit a relatively low biomass dominated by *Staphylococcus* or *Ureaplasma* species depending on the mode of neonate delivery, shifting the lung microbiome with time towards enrichment, with a more diverse mixture of oral commensals such as *Streptococcus*, *Prevotella*, *Porphyromonas*, and *Veillonella* affecting the regulation of immunoglobulins and innate immune responses [78]. An important number of lung and gut bacterial species (*Pseudomonas*, *Streptococcus*, *Prevotella*, *Fusobacterium*, *Veillonella*, *Prophyromonas*, *Neisseria*, *Haemophilus*, *Sphingomonas*, *Acinetobacter*, *Staphylococcus*, *Corynebacterium*, *Lactobacillus*, *Actinobacillus*, *Propionibacterium*, *Ralstonia*, *Megasphaera*, *Acidovorax*, *Capnocytophaga*, and *Cyanobacteria*) has been associated with lung cancer [120]. In chronic inflammatory lung diseases such as cystic fibrosis or chronic obstructive pulmonary disease (COPD), there is an outgrowth of Gammaproteobacteria like *Pseudomonas aeruginosa*, promoted by the production of reactive oxygen species during inflammation, reduced oxygen saturation, and the fermentation of mucins by commensals, which generates propionate [122,123]. PAH associated with the congenital left-to-right shunt (PAH-LTRS) is a severe disease in children. Certain bacteria like *Lactobacillus*, *Alicycliphilus*, *Castellaniella*, *Propionibacterium*, *Providencia*, *Parapusillimonas*, and *Diaphorobacter* have been seen enriched in lung PAH-LTRS patients. The *Alicycliphilus* phylum has been considered to be involved in hypoxic energy metabolism. *Lactobacillus* is reduced in the gut of PAH-LTR patients, suggesting a different role that might be related to the short-term self-healing mechanism to resist PAH-LTRS development in the early stage of lung inflammation [124]. 

In healthy subjects, the main identified fungi are usually environmental, i.e., Ascomycota (*Aspergillus*, *Cladosporium*, *Eremothecium*, and *Vanderwaltozyma*) and Microsporidia (*Systenostrema*) [125]. Respiratory fungal infection with *Aspergillus*, *Cryptococcus*, *Pneumocystis*, and endemic fungi can generate life-threatening, invasive diseases mainly in patients with compromised immune functions [126], and although antifungal drugs confer protection, drug resistance is still a severe problem [127]. Molecular mechanisms governing antifungal drug resistance occur through diverse genetic alterations, including point mutations, aneuploidy formation, and epigenetic changes given the significant plasticity observed in many fungal genomes [128]. *Aspergilli* cause pulmonary conditions like invasive aspergillosis, chronic pulmonary aspergillosis including aspergilloma and hypersensitivity pneumonitis, and allergic bronchopulmonary aspergillosis. COPD is one of many underlying risk factors for invasive aspergillosis, as the range of mortality associated with invasive aspergillosis in COPD patients is between 544,932–976,187 deaths annually [129,130]. Pulmonary mucormycosis is a relatively rare but fatal infection in immunocompromised persons and is caused by the inhalation of spores, which results in pneumonia complicated by PAH, probably due to the invasion of the perivascular tissue, resulting in vascular occlusion with subsequent necrosis and infarction of lung tissue [131]. Common fungal infections with paracoccidioidomycosis, histoplasmosis, cryptococcosis, coccidioidomycosis, aspergillosis, mucormycosis, and blastomycosis can mimic primary lung cancers, producing similar radiologic findings that are difficult to distinguish for radiologists and clinicians, who usually require a biopsy to diagnose the infectious nature of the lesions [132,133]. *Schizophyllum commune*, an environmental basidiomycete that causes respiratory system infections, allergic bronchopulmonary mycosis, sinusitis, and extremely uncommon fungal ball formation, was reported in one patient also with diabetes, hypertension, and lung cancer and was confirmed by analyzing the internal transcribed spacer, but the relationship between lung cancer and *S. commune* remains unclear [134].

Respiratory viruses include rhinoviruses and enteroviruses (*Picornaviridae*), influenza viruses (*Orthomyxoviridae*), parainfluenza, metapneumoviruses, respiratory syncytial viruses (*Paramyxoviridae*), coronaviruses (*Coronaviridae*), and several adenoviruses [135]. All possess a RNA genome except for adenoviruses, which have a double-stranded DNA genome [136]. Cellular and humoral immunity are activated in response to respiratory viral infections [137]. Respiratory viruses are related to clinical syndromes like the common cold, acute otitis media, laryngitis, sinusitis, pneumonia, bronchiolitis, influenza, and exacerbations of asthma and COPD [137]. Rhinovirus, influenza, respiratory syncytial virus, parainfluenza, adenovirus, metapneumovirus, and coronavirus cause COPD exacerbations. In cystic fibrosis patients, variable incidences of influenza A and B, respiratory syncytial virus, parainfluenza virus, rhinovirus, metapneumovirus, coronavirus, and adenovirus have been reported [138]. The association of viruses like HIV, human herpesvirus-8, hepatitis B, and hepatitis C, involving proangiogenic and pro-survival signals, with the development of PAH have been considered [139]. A potential role of possible oncogenic viruses associated with lung cancer, such as human papillomavirus (HPV), Merkel cell polyomavirus (MCPyV), and Epstein–Barr virus (EBV), is being studied. It is evident that the SARS-CoV-2 virus causes long-term lung involvement and can maintain its replication (long COVID), but its investigation is very premature, and it is uncertain what its impact on inflammation along with tumorigenic processes will be [140]. However, human lung cancer still does not show strong evidence of being associated with oncogenic viruses so far [141]. Yet, successful treatment is dependent on the early detection of various pathogens, like various aggressive pathogenic bacteria and viruses, as they may facilitate the development of an inflammatory environment prone to lung cancer initiation and progression, as well as the response to therapy, along with other factors, such as smoking habits and air pollution, among others [142]. 

## 4. Microbiome Crosstalk in the Oral-Gut-Lung Axis

The gut and lung microorganisms are interlinked by a bidirectional axis via lymphatic and blood circulation (Figure 1), dynamically regulating and maintaining the immune homeostasis of these organs, migrating immune effectors, and microbial components; therefore, the alteration of a part will impact distant epithelial cells’ bioactivities [143]. The concept of the “gut-lung axis” suggests that dysbiosis in microbiota communities’ composition, diversity, and function perturbs crosstalk with the host and can have profound influences upon immune responses and disease susceptibility, so it is associated with diverse inflammatory diseases within and outside the gastrointestinal tract and consequently involves not only host–microbe but also microbe–microbe interactions based both on localized and long-reaching effects [144,145]. In vitro and in vivo studies suggest a relevant inter-kingdom crosstalk that involves signaling pathways, physical interactions, immune response modulation, quorum-sensing molecules, production of antimicrobial agents, and nutrient exchange to maintain host homeostasis and disease evolution. The bacterial genes belong to around 150 species, represent the majority (99%) of the amplified microbiome in human stools, and are as numerous as human cells. However, recently, fungi have been recognized as an integral part of our commensal flora in the gastrointestinal ecosystem as well as the virioma, although our knowledge of it is still very limited [146]. Several combinations of fungi and bacteria have been investigated experimentally for their effect on the host [147]. One known synergistic interaction is between *Candida* and *Streptococcus*, which stimulates *Streptococcus* growth and increases biofilm formation and *Candida* pathogenicity [148]; another is between *Aspergillus fumigatus* and *Pseudomonas aeruginosa,* which increases growth related to the mold’s ability to assimilate *Pseudomonas aeruginosa*-derived volatile sulfur compounds [149]. *Helicobacter pylori* has been found within vacuoles in *C. albicans* cells, suggesting that this behavior provides an environment that *Helicobacter pylori* can use to survive the low pH of the stomach [150,151]. Exposure of poliovirus to *Bacillus cereus* led to an increase in its infectivity, supported by a higher recovery of poliovirus plaques, and its incubation with bacterial cell surface peptidoglycan and lipopolysaccharide (LPS) significantly increases yields compared to controls [152]. Influenza A virus is able to bind directly to the surface of *S. pneumoniae*, increasing the adherence of *S. pneumoniae* to human respiratory cell lines as well as other respiratory bacterial pathogens, including *Moraxella catarrhalis*, non-typable *H. influenzae*, *Pseudomonas aeruginosa*, *Staphylococcus epidermidis*, and *S. aureus*, with this last coinfection implicating alterations in the transcription and secretion of virulence factors in the pathogenicity of *S. aureus* [153,154]. A comprehensive metagenomic sequencing-based microbiome study explored colorectal cancer-associated microbiota, including bacteria, fungi, viruses, and archaea; it was able to identify 16 multi-kingdom markers, including 11 bacterial, 4 fungal, and 1 archaeal feature, which achieved good performance in diagnosing patients with colorectal cancer, suggesting the association between the biological function of several kingdoms of the gut microbiome and tumorigenic processes through a co-abundance analysis of the ecological network that revealed associations between bacterial and fungal species such as *Talaromyces islandicus* and *Clostridium saccharobutylicum* [155]. 

Oral, gut, and lung microbiota modulation might not be limited to local inter-kingdom crosstalk; it also depends on inter-compartment crosstalk between the gut and lung [146]. Oral and gut microbial dysbiosis interconnection with the inflammatory environment of the lung and other organs might be crucially involved in the pathogenesis of several diseases [5]. The bidirectional crosstalk between the oral, gut, and lung microbiomes, based on direct microbial translocation and indirect secretome effects, may develop the oral–gut–lung axis [156], through which the microbiota may also be capable of enhancing inflammation on distal lung locations via initiating the tumor-specific immune response, indeed perturbing the homeostasis of the tumor-promoting environment [143]. Surviving bacteria, cell wall fragments, and protein fragments of dead bacteria along with the cytokines and chemokines produced in the intestine go through the general circulation to enter the pulmonary circulation, leading to the activation of dendritic cells and macrophages as well as the differentiation of T cells [157]. Gut microbes might also be able to modify the anticancer response of various treatments, including chemotherapy, radiotherapy, targeted therapy, and immunotherapy, according to preclinical and clinical studies [158]. Advanced high-throughput sequencing techniques show the correspondence between specific microbiota genera and particular disease stages, allowing the design of personalized cancer treatment [159]. Therefore, the oral-gut-lung axis results from complex interactions between the different microbial components of both the gut and lung microbiotas combined with local and long-reaching immune effects (Figure 1) strongly suggest a major role in both in gastrointestinal and respiratory diseases, establishing a pre-cancerous microenvironment for cancer initiation [146]. 

## 5. Human Oral Microbiota, Inflammation, and Tumorigenesis in the Gut-Lung Axis

The human body allows the growth of a wide range of microbial communities in the oral cavity and gastrointestinal tract. The oral cavity is one of the most important interaction windows between the human body and the environment; therefore, this microenvironment has a characteristic composition that is regulated by complex signals from the host and external factors to dynamically cooperate with the host and maintain immune and metabolic status through two-way communication between the oral cavity and other organs [160]. The normally balanced, symbiotic, and generally benign commensal microbiome of the tooth-associated biofilm can undergo dysbiosis into a potentially deleterious microbiota that has been implicated in the establishment of multiple systemic diseases related to immune and inflammatory responses to pathogens virulence factors [161], initiating secondary inflammatory processes [90]. Periodontitis is a chronic non-communicable multifactorial inflammatory disease and a common oral infection with a 20–50% prevalence around the world [162] and a 72% prevalence in the Colombian population, leading to large-scale alterations in the structure of the microbial population and the functions of the entire community [163]. The interaction between oral microbiomes and the host immune system contributes to the physical structure of subgingival plaque [164]. The incidence, prevalence, and disability-adjusted life years of periodontal disease have constantly increased during the past three decades, making it the eleventh most prevalent pathology around the world [165]. Two pathogenic mechanisms can explain how a dysbiotic oral microbiota might contribute to systemic diseases. The direct mechanism states that when the epithelium lining the periodontal pockets ulcerates, it provides a direct entry point for microorganisms into systemic circulation, leading to direct effects on certain organs. The indirect mechanism is associated with the inflammatory response that is generated in response to microorganisms or their byproducts, which may have indirect systemic effects involved in the pathogenesis of chronic diseases such as cancer [166]. Several distinct original stimuli can induce inflammation in tissues, such as carcinogenic microbes, commensal microorganisms associated with a deteriorated epithelial barrier, environmental pollutants (particles and smoke), and low-grade inflammation associated with obesity, which might serve as targets to remove or neutralize for cancer prevention though vaccinations, better understanding of antibiotics usage, dietary interventions, and better environmental protection. 

The inflammatory signals regulate mesenchymal and stromal cells and mesenchymal–cancer interactions and trigger re-differentiation or stemness of post-mitotic epithelia into tumor-initiating stem-like cells or cancer stem cells (CSCs), which are essential for tumorigenesis, metastasis, and resistance to therapy through gene regulation mediated by transcription factors (TFs) like NF-kB and STAT3, increasing the proportion of CSCs among the tumor cell population and thereby elevating tumor-invasive potential [167]. The inflammation in a potential cancer site can be modulated by the microbial network either by translocation or adherence of microbes to cancer cells or by the distant release of inflammation-activating microbial metabolites as specific enzymes that enable the fermentation of nutrients like carbohydrates into short-chain fatty acids (SCFAs), which may have anti-inflammatory and immunomodulatory effects and include lipopolysaccharides, cell capsule carbohydrates, and other endotoxins [168]. The microbiome and its byproducts can travel with tumors to the site of metastasis and serve as a source of inflammation in metastasis [169]. 

SCFAs are saturated aliphatic organic acids with one to six carbon molecules that act as metabolites that are primarily produced by gastrointestinal bacteria but can also be found in periodontal pockets and regulate the inflammatory response, potentially represent a link between the microbiota and the immune system [168]. Most of the total SCFA (85%) produced in the gut is comprised of acetate, propionate, and butyrate, which upon fermentation of fibers counteract systemic inflammatory and metabolic diseases but when produced in periodontal pockets by *Porphyromonas gingivalis Treponema denticola*, *Aggregatibacter actinomycetemcomitans*, *Prevotella intermedia*, and *Fusobacterium nucleatum* act as virulence factors [170]. Acetate suppresses the accumulation of body fat and liver lipids and increases cholesterol synthesis when it is absorbed in the colon; propionate improves tissue sensitivity to insulin, which counteracts cholesterol synthesis, thus decreasing cardiovascular disease odds; and butyrate inhibits colorectal cancer cell growth [171]. Gut-derived SCFAs can affect gingival inflammation, perhaps related to an anti-inflammatory diet that decreases periodontal parameters due to fiber intake and SCFA production [172]. The oral microbe might be able to inhabit the colonic mucosa of colorectum cancer patients in biofilm-like structures containing commensal (*Parvimonas*, *Peptostreptococcus*, and *Prevotella*) and/or pathogenic (*F. nucleatum*, and *P. gingivalis*) periodontal bacteria, which could result in the establishment of a tumorigenic process [173]. The inner mucus layer in eubiosis is not inhabited by bacteria, but during intestinal dysbiosis, bacterial pathogenicity increases and downregulates MUC2 and antimicrobial peptides, leading to intestinal biofilm formation associated with enhanced microbial attachment and invasion into the colonic epithelium; inflammation through the activation of IL-6, signal transducer, and STAT3 pathways; and aberrant immune responses increasing cytotoxicity or genotoxicity, epithelial cell proliferation, and colorectal tumorigenesis [174].

Alcohol consumption and smoking reduces the relative abundance of oral *Prevotella*, *Haemophilus*, and *Neisseria* but increases *Streptococcus*, *Abiotrophia*, and *Leuconostoc*, contributing to changes in microbial metabolites, particularly SCFAs, cytokines, and chemokines, which may trigger an inflammatory response and potentially increase the risk of gastric cancer onset, changes that might be host-derived, dysbiosis-derived, or a combination of both. The reduction in levels of acetate induces lipid peroxidation following oxidative stress, resulting in gastric epithelial cell apoptosis and tumorigenesis [175]. *Helicobacter pylori* affects the structure and diversity of the oral microbiota through the interactions with oral microbes and the host to control the local environment, increasing the levels of its oral receptors MUC5B and MUC7 and leading to its retention and colonization as well as the growth and colonization of other microbes [176]. *Helicobacter pylori* has an enormous capacity to accumulate with other microbes like *F. nucleatum* and *F. periodontium* in dental plaques, with *P. gingivalis* affecting their interactions, suggesting an integrated physiological function between them [177] that could be related to superficial gastritis and metaplasia and the inhibition of the host’s immune response by *P. gingivalis* and *F. nucleatum* [176]. Autoinducer-2 is an important signaling factor generated in dental plaque that acts as a chemorepellent agent, promoting the *Helicobacter pylori* aggregates/biofilms dispersion and initiating negative chemotaxis against the signal source [178]. Infection with *Helicobacter pylori* isolated with cytotoxin-associated gene A increases LPS biosynthesis and attenuates the oral microbiota defense against microorganisms with a pathogenic potential, considerably varying the composition of *Actinomyces*, *Neisseria*, *Granulicatella*, *Helicobacter*, *Veillonella*, *Streptococcus*, *Fusobacterium*, *Haemophilus*, *Prevotella*, *Campylobacter*, *Veillonella*, *Lactobacillus*, *Stenotrophomonas*, *Serratia*, and *Roseburia* [179]. The potential mechanisms of oral microbiota in participating in the pathogenesis of gastric cancer may include an accumulation of proinflammatory bacteria (*Corynebacterium* and *Streptococcus*) and a decline in those reducing carcinogenic N-nitroso compounds (*Hemophilus*, *Neisseria*, *Parvimonas*, *Peptostreptococcus*, *Porphyromonas*, and *Prevotella*, *Haemophilus*, and *Neisseria*) [180]. 

A meta-analysis of eight cohorts and four case–control studies revealed that individuals with periodontitis are associated with a 1.71-fold increased risk for developing lung tumorigenesis, where smoking, age, and alcohol drinking status were the critical risk factors for lung cancer incidence, and chronic inflammation was a risk factor in 25% of the cases [181]. *P. gingivalis* induces the production of pro-inflammatory cytokines, including interleukin IL-1, IL-6, IL-8, TNF α, and C-reactive protein, and matrix metalloproteinases; a high serum IL-8 level predicts the subsequent diagnosis of the disease, as it was present 5 years before the diagnosis in the study [182]. IL-6 and IL-8 directly act on lung epithelium via β1 (nuclear factor of kappa light polypeptide gene enhancer in B-cells 1) pathways and induce tumorigenesis. IL-6 plays an important role in tumor initiation and progression and causes an increase in reactive oxygen species and reactive nitrogen intermediates by altering the epigenetics of certain gene and activates tumorigenic-related TFs [183]. *P. gingivalis* also acts on GRHL2, which maintains epithelial plasticity, stemness, and self-renewal capacity, inducing proliferation and metastasis by directly binding to the promoter region of RhoG, which is related to the regulation of cell shape, attachment, and mobility; it also suppresses EMT inhibiting ZEB1 promoter transactivation [184]. 

The gut microbiome network can also affect pharmacokinetics with drug metabolism, providing a natural defense against pathogenic species via competition and maintenance of the mucosa that, based on their contact with the immune system, can produce or avoid inflammatory processes [185]. The relationship between oral microbiota, inflammation, and tumorigenesis can be used to develop more specific and effective diagnostic, prevention, and treatment strategies. Early detection and monitoring of these microbiota changes could potentially allow for personalized, targeted, and faster treatments, which may improve patient outcomes and reduce the burden of colorectal, gastric, and lung cancer on healthcare systems.

## 6. Host Immunity–Microbiome Crosstalk in the Oral-Gut-Lung Axis

Host cell and microbiota interactions are fundamental for the function and development of the immune and metabolic systems, but changes in modern environments and lifestyles have led to an imbalance of this evolutionarily ancient process and have directed the establishment of immune-mediated diseases such as chronic inflammatory and tumorigenic disorders related to oral, gut, and lung microbiota dysbiosis [186]. The microbiome plays critical roles in the training and improvement of the main parts of the host’s innate and adaptive immune system, while the immune system maintains key features of host-microbe symbiosis. In a genetically susceptible host, imbalances in microbiota–immunity interactions under specific population-related environments may impact the pathogenesis of immune–inflammatory-mediated disorders [187]. New therapeutic avenues must be based on targeting either the regulatory processes, the microbiota, the barrier surfaces, or the host immune system to restore tolerance and homeostasis in the oral-gut-lung axis. The immunity–microbiome crosstalk is mainly carried out thorough the major histocompatibility complex (MHC) and toll-like receptors (TLR), which have biological functions related to health and tumorigenic processes and may translate towards future development of immune–microbiome-targeted therapeutic interventions.

### 6.1. Microbiome and Major Histocompatibility Complex (MHC) Crosstalk

**Figure 2:**
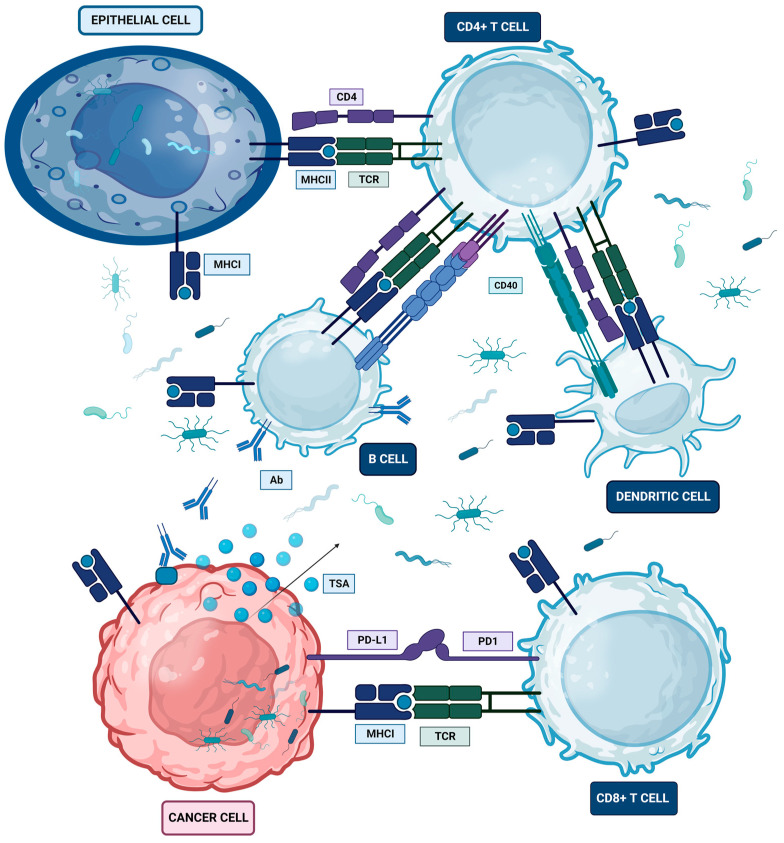
Microbiome and MHC crosstalk in healthy and tumor cell. Created with BioRender.com.

The MHC genes code for immune receptors expressed by host cells that can recognize different types of microorganisms and their secreted bioactive compounds to pass them into blood and lymphatic circulation, to induce the systemic cytokines that eventually affect oral-gut-lung axis function, and to control host’s gut microbiome composition, directly influencing the relationship between host genotype (genome) and microbiota (environment) [188]. MHC class I (MHC-I) (HLA-A, HLA-B, and HLA-C) molecules are expressed on somatic cells surfaces to generally recognize intracellular microbes (all viruses; bacteria: *Listeria monocytogenes*, *Chlamydia trachomatis*, *Coxiella burnetii*, *Mycobacterium tuberculosis*, and *Salmonella enterica*; and fungi: *Candida albicans*, *Cryptococcus neoformans*, *Aspergillus fumigatus*, and *Paracoccidioides brasiliensis*) [189,190], which promote their entry into host cells, including non-professional phagocytes such as epithelial and endothelial cells, where they may follow diverse vacuolar or cytosolic pathways [191]. MHC-I molecules also bind peptides derived from host cell’s expressed genes to transport and display this antigenic information on the cell surface, allowing CD8+ T cells to identify tumorigenic cells that are expressing mutated proteins (Figure 2); then, tumorigenic cells evolve epigenetic mechanisms like DNA and histone methylation as well as histone acetylation and deacetylation to silence and activate transcription to avoid elimination by CD8+ T cells to be able to arise and progress. MHC-I molecules are not essential for cell survival, so cancers can evade immune control by downregulation of MHC-I antigen presentation mechanisms, also frustrating immunotherapies based on re-stimulating anti-tumor CD8+ T cells, such as checkpoint blockade [192]. MHC class II (MHC-II) (HLA-DP, HLA-DQ, and HLA-DR) molecules are present on antigen-presenting cells (B cells and dendritic cells) (Figure 2) mainly for recognizing extracellular microbes (*Staphylococcus aureus*, *Streptococcus pyogenes*, *Pseudomonas aeruginosa*, and *Escherichia coli*) [189], which use virulence mechanisms to evade the antimicrobial capabilities of humoral immunity and phagocytosis, thus promoting extracellular multiplication [193]. However, there are extracellular pathogens that use an initial intracellular phase to replicate or survive within cells (bacteria: *Staphylococcus aureus*, *Streptococcus pyogenes*, *Streptococcus pneumoniae*, *Neisseria meningitidis*, *Yersinia* spp., *Pseudomonas aeruginosa*, *Cryptococcus neoformans*, *Escherichia coli* pathovars, adherent-invasive and uropathogenic *Bordetella pertussis*, and *Helicobacter pylori*; and fungi: *Histoplasma capsulatum*) [191]. MHC class II molecules can be specialists or generalists, initiating effective immune response against a relatively few or a broad range of pathogens, being the second most prevalent in human populations of microbiota-rich areas [194]. MHC class II molecules are also expressed by lung and intestinal epithelial cells under inflammatory conditions (Figure 2), suggesting that may have primary immune functions that affect the balance between tolerance and inflammation and may also be in communication with the lung from the gut directed by its microbiome [195] and involved in the activation of effector CD4+ T cells, modulating inflammatory responses related to immune enhancing or immunosuppressive activities and evocating the crosstalk that promotes epithelial renewal [196]. Secretion of tumor-specific antigen (TSA) facilitates (1) the direct recognition of MHC-II-negative cancer cells by CD4+ T cells and tumor-specific CD8+ T cells of intracellular TSA presented as peptides by MHC-I molecules on cancer cells, while B cells secrete tumor-specific antibodies (Ab) that specifically bind to TSA expressed on cancer cells, and (2) the indirect recognition of tumor-specific CD4+ T cells of cancer cells that lack MHC-II molecules by tumor-infiltrating MHC-II-positive antigen-presenting cells, which endocytose, process, and present TSA as peptides on their MHC-II molecules to CD4+ T cells [197]. The fact that the immune system has the potential to control and/or eliminate cancers even after they have become clinically evident is based on immunotherapies such as checkpoint blockade, where patients are treated with antibodies that block negative regulatory molecules such as PD-1/PD-L1 or CTLA4, which normally restrain T-cell responses, strengthening patient’s anti-tumor T-cell responses, shrinking tumors or even curing some patients. Unfortunately, the majority of clinically evident cancers almost always continue to progress, and a majority fail to respond and/or be eliminated by checkpoint blockade immunotherapy [198]. Therefore, it is important to understand how cancers evade immune control to develop mechanisms to improve immunotherapy.

Colorectal cancer has various patterns and underlying mechanisms of tumor HLA alterations. T cells and HLA-B/C antigens play an important role in controlling colorectal cancer growth, and the upregulation of HLA-B/C may trigger or enhance T-cell immunity [199]. The MSS group shows a high incidence of loss of HLA heterozygosity at chromosome 6 and 15 and coordinated transcriptional downregulation of the HLA-I heavy chain, β2-microglobulin, and antigen-presenting machinery genes, which correlate with fewer tumor-infiltrating lymphocytes, and the encapsulated phase, where the tumor is composed only of HLA-I negative cells with lymphocytes located in the stroma. Meanwhile, the MSI-H subgroup, representing consensus molecular subtype 1 with MSI and BRAF mutations, generates HLA-I losses through an accumulation of mutations in the HLA and β2-microglobulin genes with a characteristic pattern of T-cell infiltration [200]. MSI-H has a higher frequency of HLA-DR-expressing tumor cells in comparison to MSS due to unknown epigenetic mechanisms; being the predominant HLA-II molecule expressed in colorectal cancer, it is related to T-cell infiltration intensity and better prognosis and in adenomatous polyps increases with dysplasia degree [201]. MSS and hereditary MSI-H tumors that express lower levels of HLA-DR can be upregulated by the release of IFN-γ in the tumor microenvironment by infiltrating mononuclear cells [200]. HLA-I overexpression was related to a better prognosis of overall survival and probably had little impact on recurrence-free survival, so it may become a helpful marker for the clinical decision-making process regarding gastrointestinal cancer treatment and outcomes [202]. The frequency of HLA-I deficiency (≥1% tumor cells) is significantly higher in MSI tumors (52%) compared with EBV-positive tumors (23%) and the other tumors (28%), while the PD-L1 expression level is higher in EBV-positive tumors, followed by MSI tumors. HLA-I deficiency is significantly more frequent in advanced tumors (pT2-4) than in early tumors (pT1) in MSI and non-EBV non-MSI subtypes. The degree of CD8+ T-cell infiltration is significantly reduced in HLA-I-deficient tumor areas. Therefore, HLA-I and PD-L1 should be considered a common mechanism of immune escape, especially in the MSI subtype, which could become a biomarker predicting response to immune checkpoint inhibitor therapy in gastric cancer [203]. The frequency of loss of HLA heterozygosity among advanced solid tumor colorectal cancers is between 13.5–23%, which is consistent with that in primary tumors from TCGA, with no association with clinical biomarkers (KRAS, BRAF, and MSI status) [204]. HLA-Cw5 is a risk factor for gastric cancer, whereas HLA-DRB1*15 plays a protective role in this disease [205]. The overexpression of HLA-G in exosomes is associated with immune evasion, metastasis, poor prognosis, and lower overall survival, so it may provide help with identifying benign lesions that have the potential to progress into malignant ones [206]. 

HLA-I diversity can predict durable clinical benefit in lung cancer patients treated with immune checkpoint inhibitors but fail as a predictor of response or survival [207]. HLA-A is usually downregulated on the cell surface, unlike HLA-B, but HLA-A02 might escape this downregulation mechanism because its expression has a statistically significant association with improved survival in lung cancer patients, driving MHC-I gene expression along with the effect of homozygosity at the HLA-I loci, which is more pronounced in patients with strong PDL1 expression (≥50%), as this is a key factor determining increased immune cell infiltration into tumor tissue [208]. Moreover, genomic homozygosity is linked with a worse prognosis in lung cancer patients treated with single-agent immunotherapy, so HLA-I typing could become a non-invasive and cost-effective biomarker to guide treatment personalization, as patients with HLA-I homozygosity are less likely to experience clinical benefit from single-agent anti-PD1/PDL1 therapy. Moreover, this study suggests that patients with PDL1 <50% utilize other immune evasion mechanisms, including downregulation of HLA in cancer cells. The loss of HLA heterozygosity is a focal diminishing of the ability to present neoantigens to facilitate immune evasion and subclonal genome evolution, which is under strong microenvironmental selection pressures later in tumor evolution; this occurs in 40% of NSCLC patients and is associated with a high subclonal neoantigen burden, APOBEC-mediated mutagenesis, upregulation of cytolytic activity, and PD-L1 positivity [209]. 

A constant selective immunosurveillance pressure might be able to control the process of clonal selection that many tumors undergo, reducing the subset of tumor cells that express the tumor antigens recognized by T cells arising at numerous tumor progression stages. Likewise, it may also control tumor cells that evade immune detection by acquiring deficiencies in their HLA presentation pathways and then keeping important tumor antigens within cells undetected by the immune system through a variety of mechanisms, including genetic and epigenetic changes [210]. Cancer research suggests that HLA allele diversity might be wide during the establishment of tumor processes in epithelial cells and is related to the high known diversity of HLA [211], which allows an extensive flow of microbiome network information, but it must be reduced and specialized during the progression of cancer to avoid host immune response while keeping the needed flow of microbiome network information to preserve the tumorigenic process. Thus far, there are numerous studies in different types of cells that suggest how the recognition process is completed between HLA immune receptors and the most well-known extracellular and intracellular microorganisms (Table 1); however, future studies must identify the HLA alleles related to the identification of every part of the metatranscriptome network of the microbiota related to health and disease in every individual and population to deeply elucidate the changes during the establishment and progression of cancer, specifically related to the crosstalk between immune and epithelial-cancer-related host cells and the microbiome network of environmental microorganisms, along with its biological function, to reach a profound clinical significance in defining patient diagnosis and predicting which therapy will achieve the best response. Therefore, the comparison of the functional immunogenetic variation and composition of the human microbiota might provide a further understanding of the selective pressures acting on the maintenance of certain host genetic variation, as the microbiome network is a factor driving the microbe-mediated selection of MHC genes [212].

### 6.2. Microbiome and Innate Immune Receptors Crosstalk

**Figure 3:**
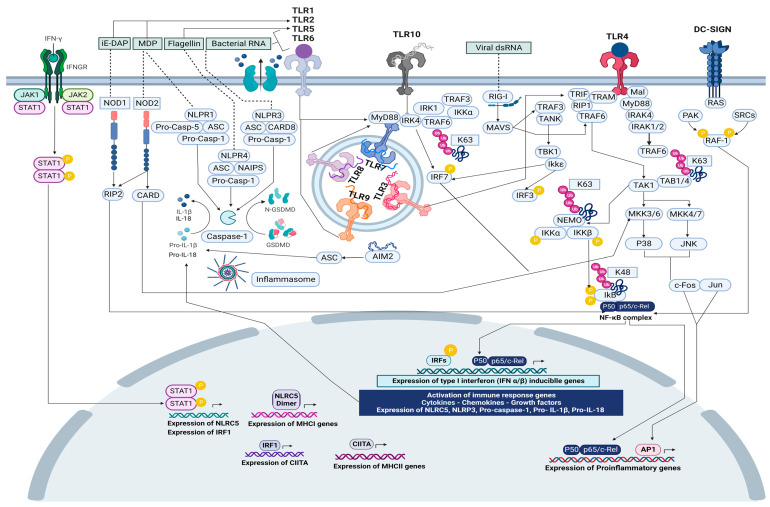
Microbiome and innate immune receptors crosstalk for the activation of signaling pathways involved in MHC, IFN, and proinflammatory gene expression. Created with BioRender.com.

The body activates innate immunity by recognizing the molecules unique to microorganisms and that are not associated with human cells, which are called pathogen-associated molecular patterns or PAMPs; these bind to pattern-recognition receptors (PRRs) on defense cells to trigger inflammatory responses. PAMPs associated with bacteria include (diacyl/triacyl) lipopeptides, lipoteichoic acids, peptidoglycan monomers, lipopolysaccharide, flagellin, and CpG DNA, along with single- and double-stranded RNA viruses [243]. PRRs recognize and bind their respective ligands and recruit adaptor molecules with the same structure through their effector domains, initiating downstream signaling pathways [244]. PRRs can be classified into five types based on protein domain homology: retinoic acid-inducible gene-I (RIG-I)-like receptors (RLRs), absent in melanoma-2 (AIM2)-like receptors (ALRs), C-type lectin receptors (CLRs), nucleotide oligomerization domain (NOD)-like receptors (NLRs), and toll-like receptors (TLRs). RLRs are a type of intracellular pattern-recognition receptor of viral and host-derived RNAs by the innate immune system, which establish an antiviral host response mediating the transcriptional induction of type I interferons and other genes, but uncontrolled RLR activity can lead to immunopathology [245]. The C-terminus has the repressor domain, which inhibits the activation, and the C-terminal domain, which regulates its own state and carried out the recognition of viral RNA, undergoing a conformational change [246]. Upon RNA binding and oligomerization, RLRs interact with the CARD domain found in mitochondrial antiviral-signaling protein (MAVS), which activates TANK-binding kinase 1 (TBK1) and the inhibitor of the nuclear factor kappaB kinase-ε (IKKε), which in turn activates IRF3 and IRF7, and along with NF-κB induces transcription of the genes encoding type I interferons and other antiviral or immunoregulatory genes (Figure 3) [245]. 

ALRs are a type of PRRs that can recognize intracellular DNA. The C-terminus is the DNA-binding domain HIN-200 that recognizes double-stranded DNA, and the N-terminus is the PYD, which binds to the PYD of apoptosis-associated speck-like protein containing CARD (ASC) (Figure 3), promoting the formation of inflammasomes and the maturation and release of IL-1β and IL-18 and regulating apoptosis related to establishment and progression of tumors [247]. CLRs are a broad and diversified family of circular structures connected by two disulfide bonds, acting as a transmembrane or secretory PRRs with phagocytic function thorough the recognition of carbohydrates on self and non-self-structures like the surface of microorganisms, mediated by carbohydrate-recognition domain (CRD), which requires calcium for interaction [248]. Transmembrane CLRs can be divided according to their topological structure into type I, where they belong to the mannose receptor family with a N-terminal that points to extracellular through at least one C-type lectin-like domain (CTLD) and multiple CRDs, and type II, where they belong to the asialoglycoprotein receptor family with a N-terminal that points to intracellular domain and only one CRD [249]. Dendritic cell-associated C-type lectin (Dectin)-1 is a type II transmembrane protein expressed in dendritic cells, macrophages neutrophils, and monocytes, with an CTLD extracellular region and an intracellular tail connected to an immunoreceptor tyrosine-based activation (ITAM) motif, indicating that the receptor also has a signal transduction function [250]. Dectin-1 can identify several fungi like yeast, *Candida albicans*, *Pneumocystis carinii*, *Cryptococcus*, and *Aspergillus* [251,252]. Dectin-2, which is different from Dectin-1, does not contain the ITAM sequence and thus has no signal transduction function and mainly recognizes α-mannan in the fungal cell wall and recognizes the *Schistosoma mansoni* egg antigen, *Mycobacterium tuberculosis* mannose-capped lipoarabinomannan, and lipoglycans from other bacterial species [253]. Another CLR, i.e., DC-specific ICAM3-grabbing non-integrin (DC-SIGN or CD209), induces signaling pathways through the activation of protein kinases or phosphatases, and its cytoplasmatic domain interacts directly or indirectly with a wide range of pathogens through mannose and fucose recognition (Figure 3). The interaction of DC-SIGN with mannose-containing pathogens such as *Mycobacterium tuberculosis*, *Mycobacterium leprae*, HIV-1, measles virus, and *Candida albicans*, modulates TLR-induced gene expression at the transcriptional or post-transcriptional level [254]. DC-SIGN triggering activates the serine/threonine protein kinase RAF1, which induces the phosphorylation of the NF-κB subunit p65 at ser276 [255]. 

CLRs are expressed on macrophages, dendritic cells, and certain tissue cells, participating in cell–cell adhesion, immune response to pathogens, and apoptosis, which is expressed by dendritic cells, which recognize PAMPs, the modified endogenous damage-associated molecular patterns (DAMPs) formed during apoptosis or from endogenous “altered self” moieties including the tumor-associated molecular patterns (TAMPs) [256]. CLRs-PAMPs binding places microorganisms in cytoplasmic vesicles for direct digestion and elimination to control infection. Following a microorganism binding or specific carbohydrate structures through the carbohydrate-recognition domain, CLRs trigger immune response signaling pathways that induce the expression of inflammatory mediators like specific cytokines that determine T-cell polarization fates, activate nuclear factor-κB, and affect signaling by toll-like receptors, supporting phagocytosis and antigen presentation, thus linking innate and adaptive immunity and maintaining physiological homeostasis, which may play role during tumorigenesis, and also eliminating cancer-related cells through their interaction with specific tumor antigens, the activation of immune surveillance and apoptosis, or by supporting tumor growth by modulating angiogenesis and trans-endothelial migration of circulating tumor cells and offering strategies for tumor escape [256].

NLRs are intracellular PRRs with three domains: (1) central nucleotide-binding domain NACHT, which is synthesized by the NLR members NAIP, CIITA, HETE, and TP1; (2) LRRs at the C-terminus, which identify ligands; and (3) the N-terminal effector domain, which is the protein interaction domain, dividing the NLRs family into five subfamilies: the NLRC subfamily with a caspase activation and recruitment domain CARD, the NLRP subfamily with a pyrin domain (PYD), the NLRB subfamily (B for BIRs) with baculovirus inhibitor of apoptosis protein repeats, the NLRA subfamily with acidic activation domains, and the NLRX subfamily with other NLR effector domains. Nucleotide-binding oligomerization domain-containing protein 1 (NOD1) recognizes the diaminopimelic acid (γ-D-glu-meso-diaminopimelic acid (iE-DAP)) of the cell wall of Gram(−) bacteria [257]. Once PAMPs directly or indirectly bind to the LRRs, the NLR molecule exposes the NACHT oligomerization domain, which triggers oligomerization activating the NLR molecule, exposing the N-terminal effector domain; then, it initiates the signal transduction [258]. NOD1 and NOD2 are activated by iE-DAP and muramyl dipeptide (MDP) in all bacterial cell walls, leading to the recruitment of receptor-interacting serine/threonine kinase (RIP2) through its CARD and the ubiquitination of the essential modulator of NF-κB and activation of the IKK (IκB kinase) complex, which phosphorylates the inhibitor of kappaB (IκBα), leading to the release of NF-κB, which, after translocation to the cell nucleus, binds to kappaB elements, thus activating pro-inflammatory cytokines. NOD1 and NOD2 also interact with the NLRP3 inflammasome, which leads to caspase-1 activation and IL-18 and IL-1β production (Figure 3). Moreover, activation of NOD1 and NOD2 leads to the formation of TBK1-IKKε complex, and the interaction of NOD2 with mitochondrial antiviral signaling protein MAVS leads to the expression of type I IFNs [259]. IFNγ induces MHC-I expression via IRF1 binding to the promoter of NLRC5, an NLR family member, as well as MHC-II transcription, inducing STAT1 and IRF1 binding to promoter IV of MHC class II transactivator (CIITA), a non-DNA-binding master regulator [260]. 

Toll-like receptors (TLRs) are type I transmembrane glycoproteins expressed on the membranes of leukocytes (dendritic cells, macrophages, and natural killer cells) and are adaptive to immunity T cells and B cells and non-immune cells (epithelial and endothelial cells and fibroblasts) [261] to bind specifically to a ligand to activate signal transduction related to immune cells response and the consequent transcription of genes to produce and secrete a variety of pro-inflammatory and antiviral factors [262]. Ten functional TLRs have been found in humans that recognize PAMPs in different cellular localization and structures, which determines the types of ligands and the recognition mechanism. TLR1, 2, 4, 5, 6, and 10 are expressed on the surface of immune cells in the form of heterodimers or homodimers, mainly recognizing the membrane components of microorganisms such as lipids, lipoproteins, and proteins; while TLR3, 7, 8, and 9 are expressed in the form of homodimers in endosomal compartments, which mainly recognize the nucleic acids of microorganisms (Figure 3). The TLR extracellular region has leucine-rich repeats (LRRs) for the recognition of specific ligands, while the intracellular domain has the same toll/IL-1R (TIR) domain as IL-1R for conducting signals transduction by binding to different receptor adaptor proteins in the cytoplasm and following myeloid differentiation factor 88 (MyD88)-dependent or -independent pathways [263]. MyD88 has a TIR domain at the C-terminus that binds to the intracellular TIR domain of TLRs and a death domain at the N-terminus that recruits IL-1R-related kinase 4 (IRAK4) and activates IRAK1 and IRAK2 through autophosphorylation of its central kinase domain and is the linker molecule in most TLR signal transduction pathways (Figure 3). Then, ubiquitin ligase TNF receptor-associated factor 6 (TRAF6) is recruited to form a complex with transforming growth factor (TGF)-β-activated kinase 1 (TAK1) and two TAK-binding proteins (TAB1 and TAB4). TRAF6 is degraded due to its own ubiquitination. The TAK1–TAB1–TAB4 complex activates the IκB kinase (IKK) complex through phosphorylation and degrades itself by ubiquitination. NF-κB is released and translocated to the nucleus, thereby regulating the transcription of inflammatory genes and the production of pro-inflammatory cytokines such as tumor necrosis factor (TNF), IL-6, IL-1, and chemokines [244]. The recognition of dsRNA by TLR10 recruits MyD88, thereby transducing signals and inhibiting interferon regulatory factor 7 (IRF7)-dependent type I interferon (IFN) production [264].

In recent years, the increased research on the recognition and binding of PRRs and their ligands related to multiple microorganisms (Table 2) has bettered the understanding of different PRRs signaling pathways and provided ideas for the treatment of immune-related diseases [244], evidencing higher frequencies of the TLRs in colorectal cancer [265], TLRs as potential targets for immunotherapy to gastric cancer [266], and TLRs with different roles in lung cancer [267]. Significant advances have been made in tumor biology to understand how TLRs play key roles in anti-cancer immunity, where exogenous and endogenous ligands are essential, but there are no TLR-stimulating therapies yet. It is currently unclear which TLR–ligand pairs will produce the desired oncological outcomes since cancers are heterogeneous; plus. the hallmarks of cancer are multi-faceted and often require multiple stimuli to generate tumors. More studies are needed to unravel the roles of TLRs in cancer considering TLR types, expression level, mutagenesis, roles of TLR adaptors, and many other factors [268].

### 6.3. The Activation of Intracellular Transcriptional Regulatory Program

The alterations in host gene expression modify microbiome composition, while changes in microbiome composition can also cause variations in host gene expression; therefore, the questions that must be answered include how, where, and when these alterations occur in each direction in order to understand the complex tumorigenic process etiology and eventually design therapeutics that target the microbiome. As was seen in the previous section, the functional immunogenetic variation of immune receptor genes confers differential microorganisms-mediated recognition; furthermore, this selection directly affects individual fitness, which might coevolve within the host and microbiome transcriptional networks to maintain the functional immunogenetic variation within human populations for detecting the microbiome, stopping pathogenic microbes’ proliferation, and preserving beneficial microbes that develop important mutualistic and commensal interactions [269].

Host immunity and energy metabolism are affected by the microbiome, as evidenced by changes in host gene expression indirectly through the activation of host microRNAs (miRNA), which repress transcribed mRNAs in human cells, triggering their degradation or inhibiting their translation [270]. Bacteria like *Listeria monocytogenes*, *Salmonella typhimurium*, and *Helicobacter pylori* can boost host miRNAs to inhibit the immune response and apoptotic signals and to increase autophagy and can also release virulence factors like effector proteins, which can inhibit host cellular pathways though host proteins binding and enhance or repress host genes expression [271]. The human microbiome has evidence that can also affect other epigenetic modifications like DNA methylation when comparing colonic tissue with different microbiota as well as histone post-translational modifications like acetylation, which is influenced by shifts in SCFAs induced by diet changes and other bacterial metabolite levels in the host [272,273]. Gene expression is also associated with changes in host chromatin accessibility and transcription factor (TF) binding induced by the exposure to specific microbiota, something evidenced in an ATAC sequencing experiment in a colonic epithelial cell co-culture model [274], suggesting that the intestinal epithelium cells could have evolved a chromatin structure and function that requires a certain microbiome to activate correctly. Therefore, the microbiome network might be able to drive the epigenetic transformation of cancer-related cells even in the absence of smoking carcinogens. It is important to establish if there are different methylation patterns in every type and subtype of gastric, colon, and lung cancer or if there is one methylation pattern that could be related to the characteristic TRN.

The flow of environmental or extracellular signals begins with its interaction with a cell transmembrane receptor that becomes activated and triggers subsequent intracellular signal transduction pathways (Figure 3), including a series of post-translational modifications in regulatory proteins like phosphorylation by protein kinases, of which hyperactivity, malfunction, and overexpression are related to tumorigenic processes [275]. The activation and deactivation of TFs by phosphorylation through the signal transduction pathway has been widely studied, showing that they can change their binding activity to target genes that encode specific proteins in response to certain extracellular signals, suggesting a key function during host–microbiome interactions in tumorigenic process. Several TFs have been related to the establishment of the characteristic abnormal patterns of gene expression in tumors, serving as integration centers of the different signal transduction pathways controlling gene networks involved in the acquisition of the hallmarks of cancer [37,38,49]. The regulatory function of TFs depends on the formation of regulatory complexes with other TFs and cofactors that cooperate to bind directly to regulatory regions like enhancers and promoters, facilitating or inhibiting the recruitment of transcriptional and regulatory machinery, including other epigenetic mechanisms [276]. Cooperative binding events are highly conserved evolutionarily and have a greater impact on gene expression compared with individual binding events because are mostly related to essential eukaryotic control processes such as the cell cycle [277] and cell-fate determination [278]. Constitutive and induced transcription of MHC-I and MHC-II genes is mediated by a set of conserved regulatory elements in their promoters and interacting TFs (Figure 3). Deviations in regulatory and epigenetic mechanisms involved in the transcriptional control of MHC genes under pathological conditions such as in cancer can become targets for pharmacological treatment of the TFs and the enzymes that modify DNA and histones [279]. All strategies targeting the regulation of TFs expression and function in current therapies might be able to lead to the selective killing of tumor cells, as normal cells tolerate the loss of TFs due to redundancy in normal signaling pathways [280]. Therefore, the TFs inside the transcriptional regulatory network (TRN) of lung and gut cancers must be identified with experimental and secondary transcriptomic analysis of normal and tumor cells in patients, considering the impact of the microbiome network in the molecular and gene regulatory metafirm of the oral-gut-lung axis. Moreover, the regulatory mechanisms related to the acquisition of the hallmarks of cancer in tumor cells must be analyzed globally from the TRN perspective using genomics, epigenomics, and transcriptomics analysis of lung and gut cells.

## 7. Conclusions

The massive complexity of the that trans-kingdom crosstalk between the microbes in the oral-gut-lung axis and the host epithelial and immune cells is immense. Several studies have been developed to analyze the effect of the microbiome in tumor processes. However, no study has analyzed the stimuli events of the microbiome as a network that triggers the inflammatory response as correlated to its communication with the TRN of cancer-related cells in order to understand the global tumorigenic process in the system’s biology context. Moreover, most of the studies have focused on one specific organ rather than an inter-organ microbiome axis in the establishment and progression of inflammatory, immune, and tumorigenic processes through local and systemic dysbiotic phenomena. Therefore, the regulatory proteins and the epigenomic regulatory mechanisms related to its expression can be targeted for therapy, thus modulating the signaling events and signal transduction hubs downstream in early tumor initiation as well as in metastatic spread and outgrowth. Additionally, a better understanding of the underlying mechanisms leading to the development of CSCs by the specific cancer transcriptional regulatory network may lay the foundations for precise measures able to prevent the onset of cancer from the establishment of inflammatory processes. Therefore, novel bioinformatics pipelines and computational methods need to be developed that integrate the multiple high-throughput sequencing datasets (metagenomics, metatranscriptomics, genomics, transcriptomics, and epigenomics) to discover complex system biology process involved in the changes in the regulation of gene expression of host cells mediated by the microbiome composition (bacteria, viruses, and fungi). This research will help elucidate the key information to overcome the challenges and take advantage of the full potential of the oral-gut-lung axis microbiota and develop anti-cancer therapies based on a multiomics approach that incorporates microbial modulation therapy and epidrugs in the control of immune and tumor-related genes expression as well as the communication between host microbiome and epithelial and immune cells in the oral-gut-lung axis.

## Figures and Tables

**Table 1:** Microorganisms and their HLA/MHC immune-related receptors in different cell types. Allele number (*).

Microorganism	Immune Receptor (IR)	References
*Listeria monocytogenes*	HLA-C_HLA-DR	[213]
*Chlamydia trachomatis*	HLA-A2_HLA-DRB1_HLA-DQB1	[214]
*Coxiella burnetii*	HLA-A_HLA-B_HLA-DRB1	[215]
*Mycobacterium tuberculosis*	HLA-E_HLA-DRB5*2_HLA-DRB1*14	[216,217]
*Salmonella enterica*	HLA-E_HLA-B27	[218,219]
*Staphylococcus aureus*	HLA-A_HLA-B_HLA-DRB1_HLA-DRA	[220,221]
*Streptococcus pyogenes*	HLA-B6_HLA-DR4_HLA-DQA1_HLA-DQ8	[222,223]
*Neisseria meningitidis*	HLA-DRB	[224]
*Streptococcus pneumoniae*	HLA-A_HLA-B_HLA-DR_HLA-DRB4	[225,226,227]
*Pseudomonas aeruginosa*	HLA-DRA_HLA-DRB3_HLA-DQ	[228,229,230]
*Cryptococcus neoformans*	HLA-A_HLA-B_HLA-C_HLA-DQB5	[231]
*Helicobacter pylori*	HLA-CW*03_HLA-DRB1*01_HLA-DQA1_HLA-DQB1_HLA-A*2	[232,233]
*Candida albicans*	HLA-DR_HLA-DRB1_HLA-DPA1	[234,235,236]
*Cryptococcus neoformans*	HLA-A_HLA-DRA_HLA-DRB5_HLA-DQB1	[231,237]
*Aspergillus fumigatus*	HLA-A2	[238,239]
*Paracoccidioides brasiliensis*	HLA-DR	[240]
*Histoplasma capsulatum*	HLA-A_HLA-B_HLA-DR_HLA-DQ_HLA-DP	[241,242]

**Table 2:** Toll like receptors (TLRs), their ligands related to microorganisms, and the localization of the tumorigenic process.

Immune Receptor (IR)	Microorganism–Ligand	Localization
TLR1	Bacteria–Triacyl lipopeptide, lipoteichoic acid, and peptidoglycans Gram(+) bacteria–Lipopolysaccharide Fungi–Zymosan	Lung carcinoma Metastatic colorectal cancer Gastric cancer
TLR2	Bacteria–Triacyl lipopeptide, lipoteichoic acid, peptidoglycans, and diacylated lipopeptides Viruses surface GP Gram(+) bacteria–LP and PG Gram(−) bacteria–Porin and PG Fungi–Zymosan, β-glycan, and mannan Protozoa GPI anchors	Colorectal cancer Gastric cancer Lewis lung carcinoma
TLR3	dsDNA Viruses–dsRNA	Colorectal cancer Gastric cancer Lung tumoral exosomes NSCLC
TLR4	Viruses surface GP Gram(−) bacteria–Lipopolysaccharide Fungi–Mannan Protozoa–GPI anchors	Colorectal cancer Gastric cancer Lung cancer NSCLC
TLR5	Gram(−) bacteria–Flagellin	Colorectal cancer Gastric cancer NSCLC
TLR6	Gram(+) bacteria–Lipopolysaccharide	Colorectal cancer Lung cancer
TLR7	Viruses–ssRNA	Colorectal cancer Gastric cancer NSCLC
TLR8	Viruses–ssRNA	Primary lung tumors
TLR9	Viruses Gram(+)/(−) bacteria Bacterial unmethylated CpG DNA Fungi–dsDNA Protozoa–dsDNA	Colorectal cancer Gastric cancer Lung carcinoma Lung cancer PBMCs
TLR10	dsDNA	-

## Data Availability

Data sharing is not applicable to this article.

## Abbreviations

KSHV	Kaposi’s sarcoma-associated herpesvirus
HHV8	Human herpesvirus-8
EBV	Epstein–Barr virus
TRN	Transcription factor or transcriptional regulatory network
IEGs	Immediate-early genes
TFs	Transcription factors
IL-8	Interleukin 8
CXCL8	Chemokine (C-X-C motif) ligand 8
CXCL7	Chemokine (C-X-C motif) ligand 7
CCL19	Chemokine (C-C motif) ligand 19
CXCL13	Chemokine (C-X-C motif) ligand 13
MSI	Microsatellite instability
MSS	Microsatellite stability
APC	Adenomatous polyposis coli
KRAS	Kirsten rat sarcoma virus
PIK3CA	Phosphatidylinositol-4,5-bisphosphate 3-kinase catalytic subunit alpha
SMAD4	Mothers against decapentaplegic homolog 4
TP53	Tumor protein P53
DCC	Netrin receptor
SMAD	Mothers against decapentaplegic
BRAF	B-Raf Proto-Oncogene
Wnt	Wingless-related integration site
BMP	Bone morphogenetic protein
TGFβ	Transforming growth factor-beta
Ras	Rat sarcoma virus
ST6GALNAC1	ST6 N-acetylgalactosaminide alpha-2,6-sialyltransferase 1
CDH1	Cadherin 1
STK11	Serine/threonine kinase 11
MLH1	mutL homolog 1
MSH2	mutS homolog 2
EPCAM	Epithelial cell adhesion molecule
MSH6	mutS homolog 6
PMS2	PMS1 homolog 2, mismatch repair system component
FAP	Familial adenomatous polyposis
BRCA2	BReast CAncer gene 2
PALB2	Partner and localizer of BRCA2
CLDN18-ARHGAP	Fusion Gene_Claudin 18-Rho GTPase-activating protein
ARID1A	AT-rich interaction domain 1A
PD-L1/2	Programmed cell death ligand ½
ICIs	Immune checkpoint inhibitors
CTLA-4	Cytotoxic T-lymphocyte associated protein 4
PD-1	Programmed cell death 1
LAG-3	Lymphocyte activating 3
TIM-3	Hepatitis A virus cellular receptor 2
PD-L1	CD274 molecule
ERBB2	erb-b2 receptor tyrosine kinase 2
MET	Methoprene-tolerant proto-oncogene, receptor tyrosine kinase
NKX2	Ventral nervous system defective
DOK2	Docking protein 2
ALK	Anaplastic lymphoma receptor tyrosine kinase
ROS1	ROS proto-oncogene 1, receptor tyrosine kinase
RET	Ret proto-oncogene
ALK/EML4	Anaplastic lymphoma receptor tyrosine kinase–Echinoderm microtubule-associated protein-like 4
TTN	titin
CSMD3	CUB and Sushi multiple domains 3
MUT16	MUTator 16
RYR2	Ryanodine receptor 2
BRINP3	BMP/retinoic acid inducible neural specific 3
COL11A1	Collagen type XI alpha 1 chain
GRIN2B	Glutamate ionotropic receptor NMDA type subunit 2B
MUC5B	Mucin 5B, oligomeric mucus/gel-forming
NLRP3	NLR family pyrin domain containing 3
TENM3	Teneurin transmembrane protein 3
NSCLC	Non-small cell lung cancer
LUAD	Lung adenocarcinoma
LUSC	Lung squamous cell carcinoma
TCGA-STAD	The Cancer Genome Atlas Stomach Adenocarcinoma
RUNX2	Runt-related transcription factor 2
SOX4	SRY-box transcription factor 4
SOX17	SRY-box transcription factor 17
BZW2	Basic leucine zipper and W2 domains 2
FOXM1	Forkhead box M1
ZBTB16	Zinc finger and BTB domain containing 16
TAL1	T-cell acute lymphocytic leukemia 1 bHLH transcription factor 1, erythroid differentiation factor
KLF4	Kruppel-like transcription factor 4
EPAS1	Endothelial PAS domain protein 1
HOXC6	Homeobox C6
ID4	Inhibitor of DNA binding 4
KLF2	Kruppel-like transcription factor 2
MEIS1	Meis homeobox 1
NR2F1	Nuclear receptor subfamily 2 group F member 1
TBX4	T-box transcription factor 4
TCF21	Transcription factor 21
TFAP2C	Transcription factor AP-2 gamma
LMO2	LIM domain only 2
MNDA	Myeloid cell nuclear differentiation antigen
FOXF1	Forkhead box F1
HLF	HLF transcription factor, PAR bZIP family member
RFX2	Regulatory factor X2
DLX5	Distal-less homeobox 5
MYBL2	MYB proto-oncogene like 2
NR4A3	Nuclear receptor subfamily 4 group A member 3
PKNOX2	PBX/knotted 1 homeobox 2
GPRASP1	G protein-coupled receptor associated sorting protein 1
PAH	Pulmonary arterial hypertension
DEGs	Differentially expressed genes
CpG	Cytosine–guanine dinucleotides
DNMT1	DNA methyltransferase 1
DNMT3A	DNA methyltransferase 3 Alpha
DNMT3B	DNA methyltransferase 3 Beta
TET	Ten-eleven translocation family
FIGN	Fidgetin, microtubule severing factor
HTRA3	HtrA serine peptidase 3
BDNF	Brain-derived neurotrophic factor
HCN4	Hyperpolarization activated cyclic nucleotide gated potassium channel 4
STAC2	SH3- and cysteine-rich domain 2
SEPT9	Septin 9
SDC2	Syndecan 2
MGMT	O-6-methylguanine-DNA methyltransferase
NDGR4	N-Myc Downstream-Regulated Gene 4 Protein
BMP3	Bone morphogenetic protein 3
VIM	Vimentin
SFRP	Secreted frizzled-related protein
p16	Cyclin-dependent kinase inhibitor 2A
LINE-1	Long interspersed nuclear element 1
BCAT1/IKZF1	Branched-chain amino acid transaminase 1-IKAROS family zinc finger 1
RASSF1A	Ras-association domain family member 1
Wnt5A	Wnt family member 5A
SFRP2	Secreted frizzled-related protein 2
DKK2	Dickkopf WNT signaling pathway inhibitor 2
PCDH10	Protocadherin 10
TMEFF2	Transmembrane protein with EGF-like and two follistatin-like domains 2
SFRP1	Secreted frizzled-related protein 1
HS3ST2	Heparan sulfate-glucosamine 3-sulfotransferase 2
CDH1/RHOA	Cadherin 1-ras homolog family member A
RPRM	Reprimo, TP53-dependent G2 arrest mediator homolog
ZNF793	Zinc finger protein 793
RUNX3	Runt-related transcription factor 3
PTEN	Phosphatase and tensin homolog
CXXC4	CXXC finger protein 4
TIMP2	TIMP metallopeptidase inhibitor 2
COL9A2	Collagen type IX alpha 2 chain
EYA1	EYA transcriptional coactivator and phosphatase 1
ZNF365	Zinc finger protein 365
AMPH	Amphiphysin
SORCS3	Sortilin-related VPS10 domain containing receptor 3
AJAP1	Adherens junctions-associated protein 1
TGFβ2	Transforming growth factor beta 2
SHOX2	Short stature homeobox 2
RASSF1A	Ras-association domain family member 1
RASSF1A-RARβ2	Ras-association domain family member 1–retinoic acid receptor beta 2
SHOX2-PTGER4	Short stature homeobox 2-prostaglandin E receptor 4
p16-RARβ2	Cyclin=dependent kinase inhibitor 2A–retinoic acid receptor beta 2
DAL-1	Erythrocyte membrane protein band 4.1 like 3
EPHB6	EPH receptor B6
HS3ST2	Heparan sulfate-glucosamine 3-sulfotransferase 2
TMEM88	Transmembrane protein 88
ELMO3	Engulfment and cell motility 3
HOXA9	Homeobox A9
RARβ2	Retinoic acid receptor beta 2
CDH13	Cadherin 13
CDKN2A/P16	Cyclin-dependent kinase inhibitor 2A
CASP8	Caspase 8
TNFRSF6/Fas	Fas cell surface death receptor
TRAIL-R1/DR4	TNF receptor superfamily member 10a
SCLC	Small-cell carcinoma
cfDNA	Cell-free DNA
ANK1	Ankyrin 1
CCND2	Cyclin D2
GATA3	GATA-binding protein 3
KCNH5	Potassium voltage gated channel subfamily H member 5
RARβ	Retinoic acid receptor beta
RASSF1	Ras-association domain family member 1
CLDN1	Claudin 1
TP63	Tumor protein p63
TPX5	T-box transcription factor 5
ADHFE1	Alcohol dehydrogenase iron containing 1
HNF1B	HNF1 homeobox B
EZH2	Enhancer of zeste 2 polycomb-repressive complex 2 subunit
IBD	Inflammatory bowel disease
COPD	Chronic obstructive pulmonary disease
PAH-LTRS	PAH associated with the congenital left-to-right shunt
CSCs	Cancer stem cells
NF-kB	Nuclear factor kappaB
STAT3	Signal transducer and activator of transcription 3
SCFAs	Short-chain fatty acids
MUC2	Mucin 2, oligomeric mucus/gel-forming
IL-6	Interleukin 6
MUC5B	Mucin 5B, oligomeric mucus/gel-forming
MUC7	Mucin 7, secreted
IL-1	Interleukin 1
IL-8	Interleukin 8
TNF α	Tumor necrosis factor alpha-like
GRHL2	Grainyhead-like 2 transcription factor
RhoG	Ras homology growth
ZEB1	Zinc finger E-box binding homeobox 1
MHC	Major histocompatibility complex
TLR	Toll-like receptors
HLA-A	Major histocompatibility complex, class I, A
HLA-B	Major histocompatibility complex, class I, B
HLA-C	Major histocompatibility complex, class I, C
CD8+ T cells	Cytotoxic T lymphocytes
HLA-DP	Major histocompatibility complex, class II, DP
HLA-DQ	Major histocompatibility complex, class II, DQ
HLA-DR	Major histocompatibility complex, class II, DR
CD4+ T cells	Helper T lymphocytes
TSA	Tumor-specific antigen
CTLA4	Cytotoxic T-lymphocyte-associated protein 4
APOBEC	Apolipoprotein B mRNA-editing enzyme, catalytic polypeptide 3
PAMPs	Pathogen-associated molecular patterns
PRRs	Pattern-recognition receptors
RLRs	Retinoic acid-inducible gene-I (RIG-I)-like receptors
ALRs	Melanoma-2 (AIM2)-like receptors
CLRs	C-type lectin receptors
NLRs	Nucleotide oligomerization domain (NOD)-like receptors
MAVS	Mitochondrial antiviral-signaling protein
TBK1	TANK-binding kinase 1
IKKε	Nuclear factor kappaB kinase-ε
IRF3	Interferon regulatory factor 3
IRF7	Interferon regulatory factor 7
CRD	Carbohydrate-recognition domain
CTLD	C-type lectin-like domain
Dectin-1	C-type lectin 1
ITAM	Immunoreceptor tyrosine-based activation motif
DC-SIGN	DC-specific ICAM3-grabbing non-integrin
NAIP	NLR family apoptosis inhibitory protein
CIITA	Class II major histocompatibility complex transactivator
HETE	Cytochrome P450 family 4 subfamily F member 2
TP1	Transition protein 1
TIR	Toll/IL-1R domain
